# Supplementary material for: Heterocornols from the Sponge-Derived Fungus Pestalotiopsis heterocornis with Anti-Inflammatory Activity
Source: Mar Drugs. 2021 Oct 20;19(11):585. doi: 10.3390/md19110585 (PMC8620458; doi:10.3390/md19110585)
Supplement: Supplementary file 1 [file marinedrugs-19-00585-s001.zip › marinedrugs-1371309-supplementary.pdf]

Supporting Information

Heterocornols from the Sponge-Derived Fungus *Pestalotiopsis heterocornis* with Anti-Inflammatory Activity

Hui Lei [a], Xiaoxu Bi [b], Xiuping Lin [c], Jianglian She [c], Xiaowei Luo [d], Hong Niu [a], Dan Zhang [a] and Bin Yang [c],*

[a] School of Pharmacy, Southwest Medical University, Luzhou 646000, China; huilei@swmu.edu.cn; dawn123454@swmu.edu.cn；zhangdan@swmu.edu.cn

[b] College of Agriculture and Life Sciences, Kunming University, Kunming 50241, China；bxx2797@163.com

[c] CAS Key Laboratory of Tropical Marine Bio-resources and Ecology, Guangdong Key Laboratory of Marine Materia Medica, South China Sea Institute of Oceanology, Chinese Academy of Sciences, Guangzhou 510301, China

[d] Institute of Marine Drugs, Guangxi University of Chinese Medicine, Nanning 530200, China

*Correspondence: yangbin@scsio.ac.cn (B. Yang); Tel: +86-020-89023174 (B.Y.)

Figure S1. HRESI-MS spectrum of the new compound **1**

Figure S2. ^{1}H NMR (500 MHz, CD_3OD) spectrum of the new compound **1**

Figure S3. ^{13}C NMR (125 MHz, CD_3OD) spectrum of the new compound **1**

Figure S4. HSQC spectrum of the new compound **1**

Figure S5. HMBC spectrum of the new compound **1**

Figure S6. COSY spectrum of the new compound **1**

Figure S7. NOESY spectrum of the new compound **1**

Figure S8. HRESI-MS spectrum of the new compound **2**

Figure S9. ^{1}H NMR (500 MHz, CD_3OD) spectrum of the new compound **2**

Figure S10. ^{13}C NMR (125 MHz, CD_3OD) spectrum of the new compound **2**

Figure S11. HSQC spectrum of the new compound **2**

Figure S12. HMBC spectrum of the new compound **2**

Figure S13. COSY spectrum of the new compound **2**

Figure S14. NOESY spectrum of the new compound **2**

Figure S15. HRESI-MS spectrum of the new compound **3**

Figure S16. ^{1}H NMR (500 MHz, CD_3OD) spectrum of the new compound **3**

Figure S17. ^{13}C NMR (125 MHz, CD_3OD) spectrum of the new compound **3**

Figure S18. HSQC spectrum of the new compound **3**

Figure S19. HMBC spectrum of the new compound **3**

Figure S20. NOESY spectrum of the new compound **3**

Figure S21. HRESI-MS spectrum of the new compound **4**

Figure S22. ^{1}H NMR (500 MHz, CD_3OD) spectrum of the new compound **4**

Figure S23. ^{13}C NMR (125 MHz, CD_3OD) spectrum of the new compound **4**

Figure S24. HSQC spectrum of the new compound **4**

Figure S25. HMBC spectrum of the new compound **4**

Figure S26. COSY spectrum of the new compound **4**

Figure S27. NOESY spectrum of the new compound **4**

Figure S28. calculated and experimental ECD spectras of **3** and **4** using a truncated model.

Figure S29. calculated and experimental ECD spectras of **3** and **4**.

Figure S30. The data of DP4plus method of compound **3**

Figure S31. Comparison of the experimental ^{13}C NMR data and mean absolute error of compound **3**

Figure S32. $DP4^+$ probability of ^{13}C NMR chemical shifts（compound **3**）.

Figure S33. The data of DP4plus method of compound **4**

Figure S34. Comparison of the experimental ^{13}C NMR data and mean absolute error of compound **4**

Figure S35. $DP4^+$ probability of ^{13}C NMR chemical shifts（compound **4**）.

Figure S36. HRESI-MS spectrum of the new compound **5**

Figure S37. ^{1}H NMR (500 MHz, CD_3OD) spectrum of the new compound **5**

Figure S38. ^{13}C NMR (125 MHz, CD_3OD) spectrum of the new compound **5**

Figure S39. ^{135} DEPT (125 MHz, CD_3OD) spectrum of the new compound **5**

Figure S40. HSQC spectrum of the new compound **5**

Figure S41. HMBC spectrum of the new compound **5**

Figure S42. COSY spectrum of the new compound **5**

Figure S43. HRESI-MS spectrum of compound **6**

Figure S44. ^{1}H NMR (500 MHz, CD_3OD) spectrum of compound **6**

Figure S45. ^{13}C NMR (125 MHz, CD_3OD) spectrum of compound **6**

Figure S46. HSQC spectrum of compound **6**

Figure S47. HMBC spectrum of compound **6**

Figure S48. HRESI-MS spectrum of the new compound **7**

Figure S49. ^{1}H NMR (500 MHz, CD_3OD) spectrum of the new compound **7**

Figure S50. ^{13}C NMR (125 MHz, CD_3OD) spectrum of the new compound **7**

Figure S51. HSQC spectrum of the new compound **7**

Figure S52. HMBC spectrum of the new compound **7**

Figure S53. HRESI-MS spectrum of the new compound **8**

Figure S54. ^{1}H NMR (500 MHz, CD_3OD) spectrum of the new compound **8**

Figure S55. ^{13}C NMR (125 MHz, CD_3OD) spectrum of the new compound **8**

Figure S56. HSQC spectrum of the new compound **8**

Figure S57. HMBC spectrum of the new compound **8**

Figure S58. HRESI-MS spectrum of the new compound **9**

Figure S59. EI-MS spectrum of the new compound **9**

Figure S60. ^{1}H NMR (500 MHz, $CDCl_3$) spectrum of the new compound **9**

Figure S61. ^{13}C NMR (125 MHz, $CDCl_3$) spectrum of the new compound **9**

Figure S62. HSQC spectrum of the new compound **9**

Figure S63. HMBC spectrum of the new compound **9**

Figure S64. HPLC chromatograms of EtOAc extracts monitored

Mass Spectrum SmartFormula Report

Analysis Info		Acquisition Date	12/5/2018 4:00:09 PM	
Analysis Name	D:\Data\MS\data\201812\yangbin_L32_neg_21_01_5869.d			
Method	LC_Direct Infusion_pos_70-500mz.m	Operator	SCSIO	
Sample Name	yangbin_L32_neg	Instrument	maXis	255552.00029
Comment				

Acquisition Parameter

Source Type	ESI	Ion Polarity	Positive	Set Nebulizer	0.4 Bar
Focus	Active	Set Capillary	4500 V	Set Dry Heater	180 °C
Scan Begin	70 m/z	Set End Plate Offset	-500 V	Set Dry Gas	4.0 l/min
Scan End	1500 m/z	Set Charging Voltage	0 V	Set Divert Valve	Waste
		Set Corona	0 nA	Set APCI Heater	0 °C

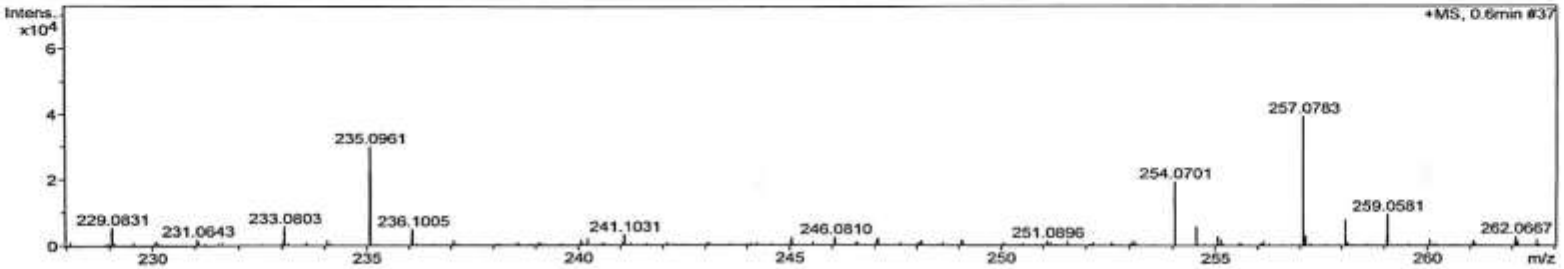

Meas. m/z	#	Ion Formula	Score	m/z	err [ppm]	err [mDa]	mSigma	rdb	e⁻ Conf	N-Rule
235.0961	1	C13H15O4	100.00	235.0965	-1.5	-0.4	14.2	6.5	even	ok
257.0783	1	C13H14NaO4	100.00	257.0784	0.7	0.2	33.1	6.5	even	ok

yangbin_L32_neg_21_01_5869.d
Bruker Compass DataAnalysis 4.1 printed: 12/5/2018 4:11:57 PM by: SCSIO Page 1 of 1

Figure S1. HRESI-MS spectrum of the new compound **1**

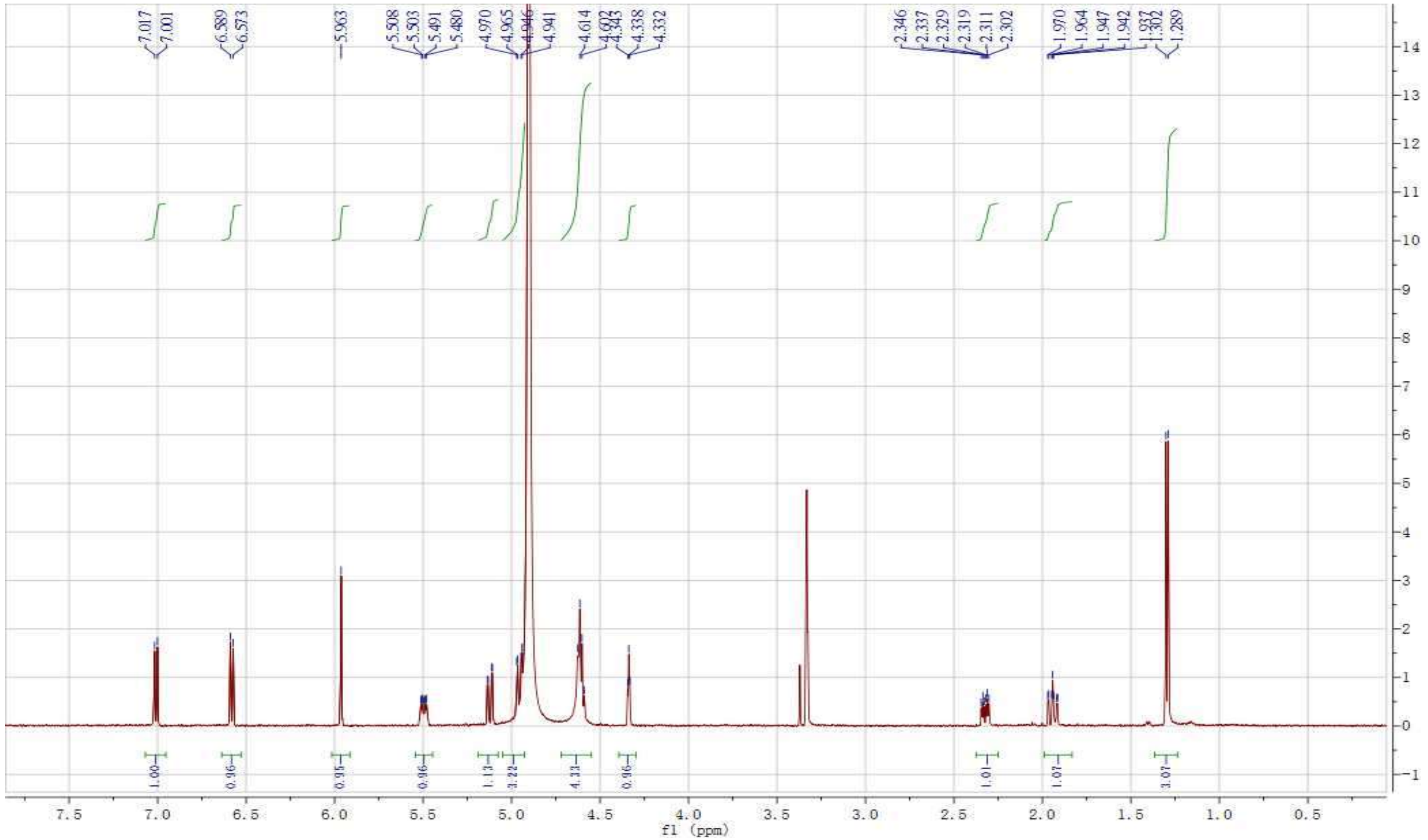

Figure S2. ^{1}H NMR (500 MHz, CD_3OD) spectrum of the new compound **1**

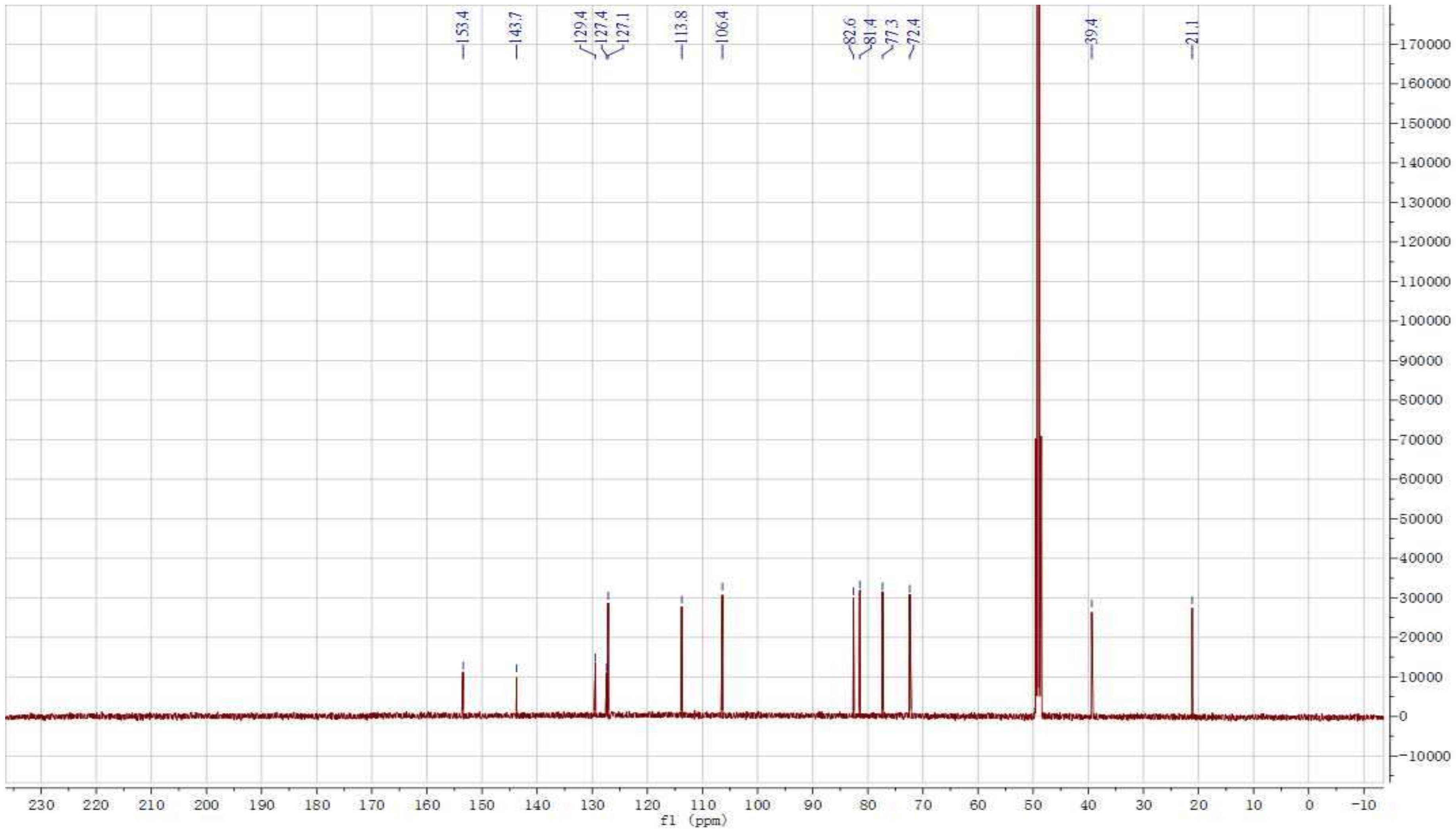

Figure S3. ^{13}C NMR (125 MHz, CD_3OD) spectrum of the new compound **1**

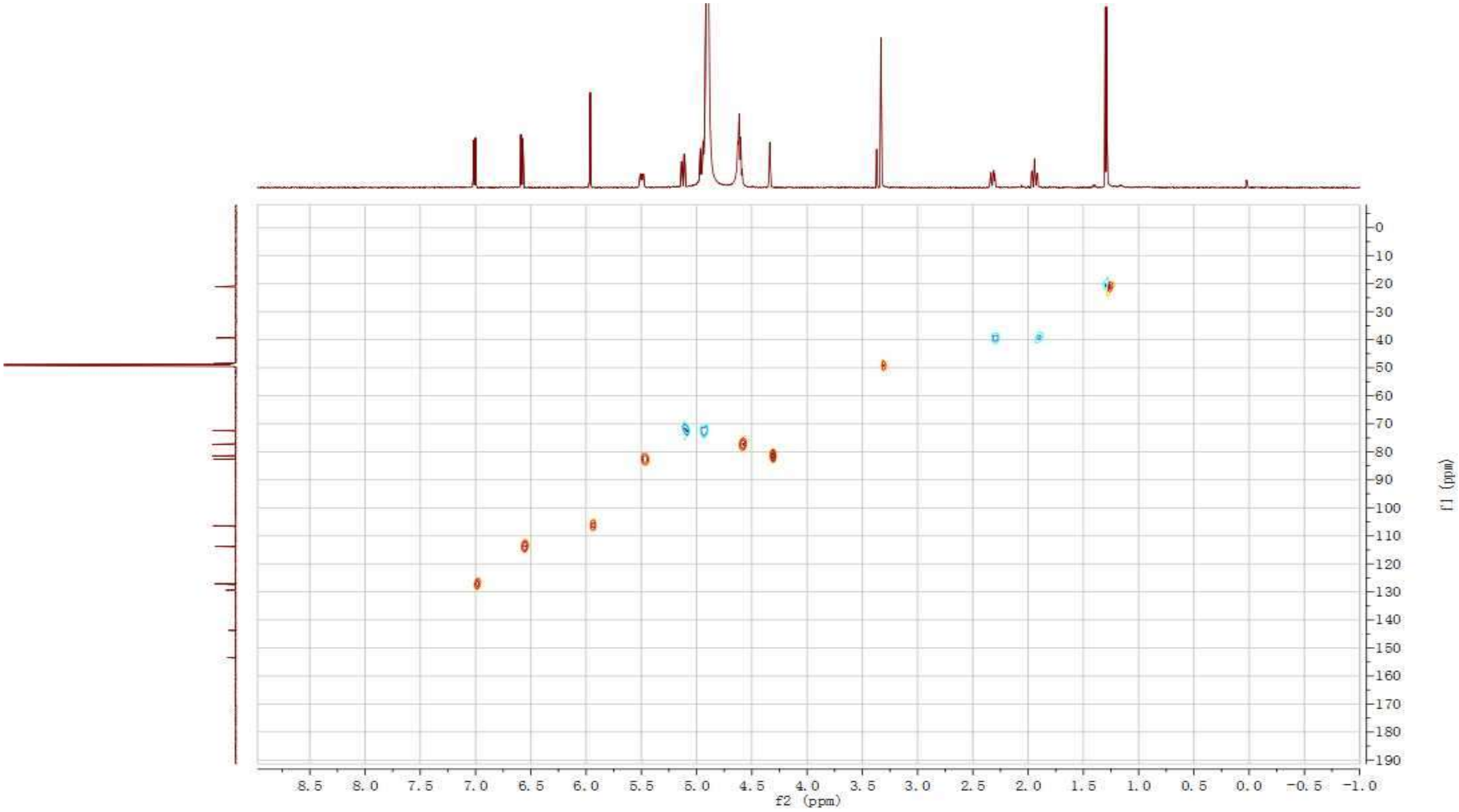

Figure S4. HSQC spectrum of the new compound **1**

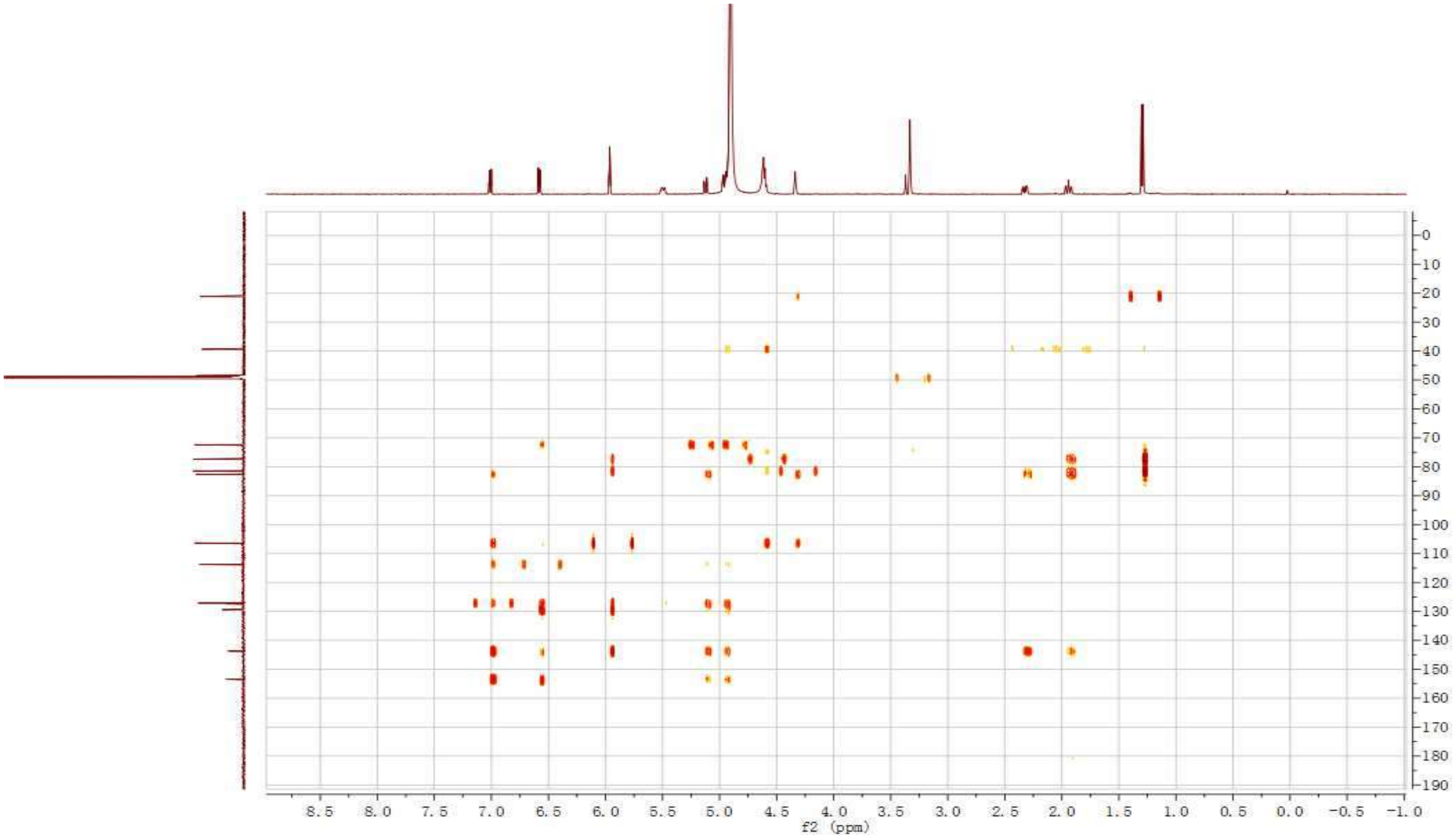

Figure S5. HMBC spectrum of the new compound **1**

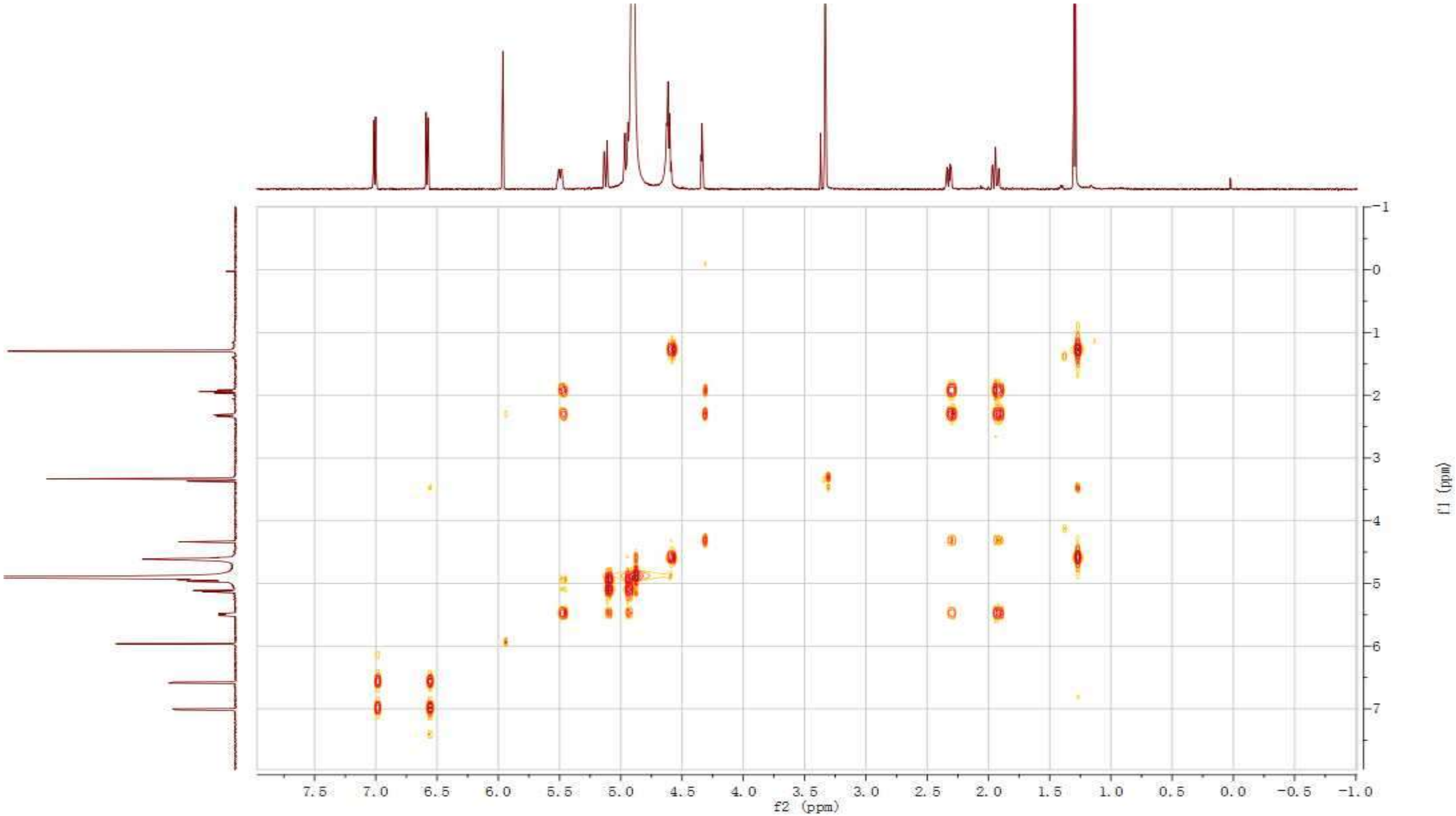

Figure S6. COSY spectrum of the new compound **1**

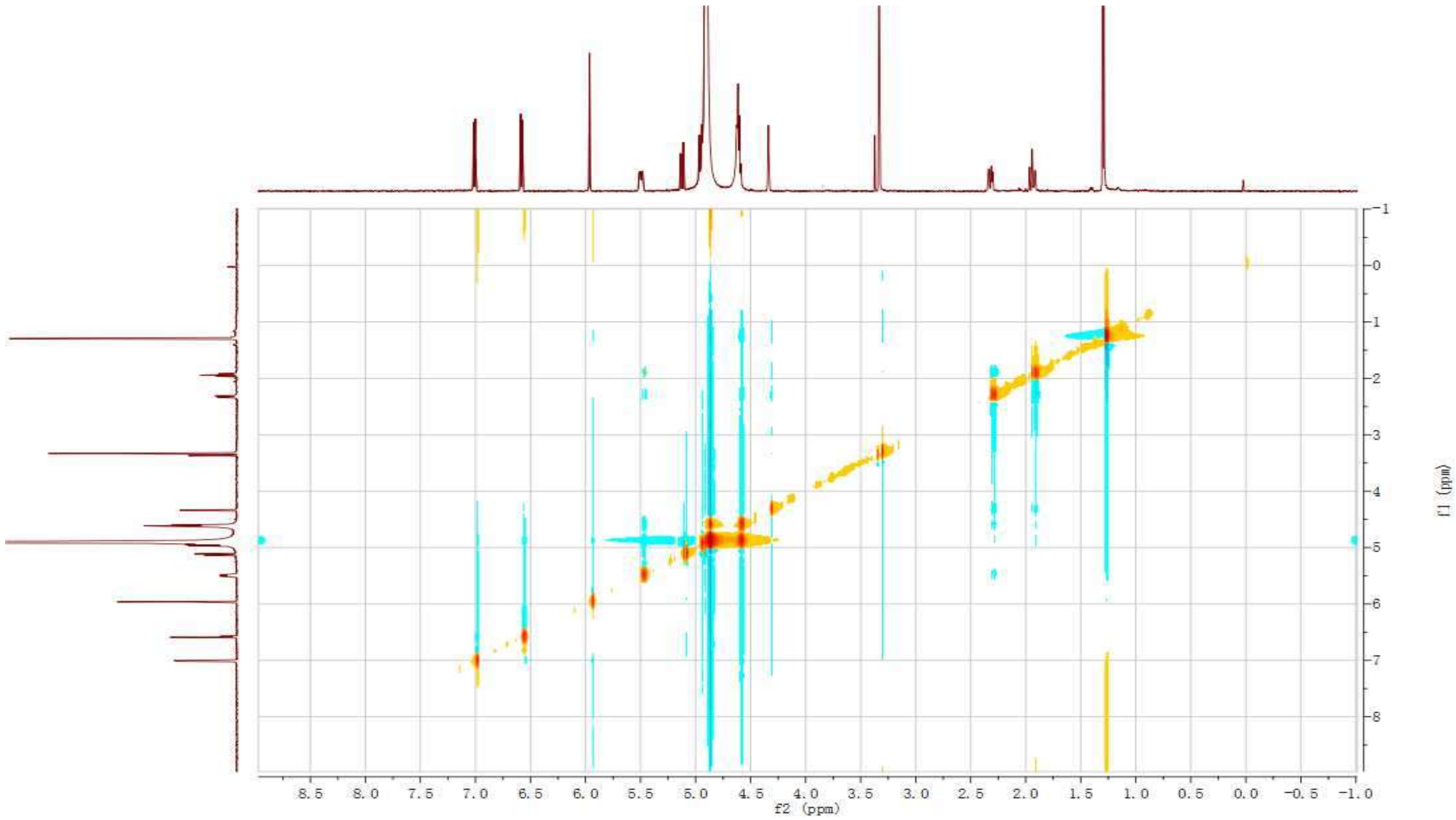

Figure S7. NOESY spectrum of the new compound **1**

HR-ESI-MS-NEG

Mass Spectrum SmartFormula Report

**Analysis Info**		Acquisition Date	8/14/2019 2:59:51 PM	
Analysis Name	D:\Data\MS\data\201908\wanjianjiao_LH-H1_neg_41_01_7090.d			
Method	LC_Direct Infusion_pos_70-500mz.m	Operator	SCSIO	
Sample Name	wanjianjiao_LH-H1_neg	Instrument	maXis	255552.00029
Comment				

Acquisition Parameter

Source Type	ESI	Ion Polarity	Positive	Set Nebulizer	0.4 Bar
Focus	Active	Set Capillary	4500 V	Set Dry Heater	180 °C
Scan Begin	70 m/z	Set End Plate Offset	-500 V	Set Dry Gas	4.0 l/min
Scan End	1500 m/z	Set Charging Voltage	0 V	Set Divert Valve	Waste
		Set Corona	0 nA	Set APCI Heater	0 °C

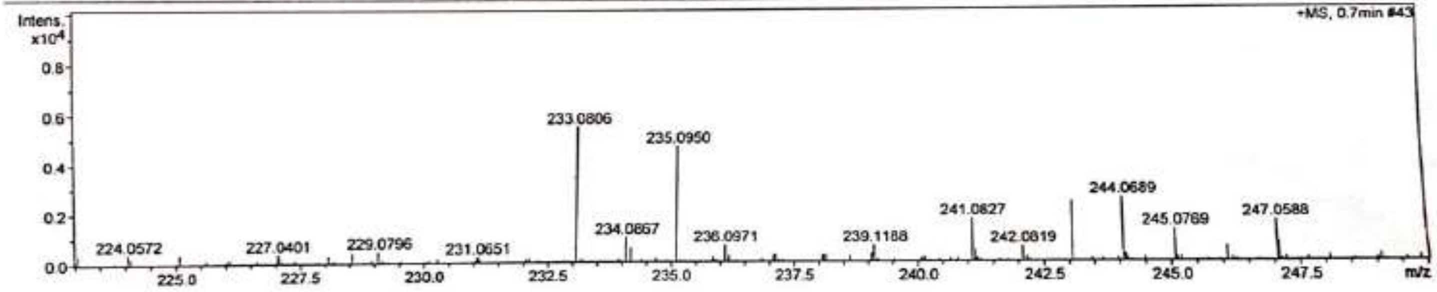

Meas. m/z	#	Ion Formula	Score	m/z	err [ppm]	err [mDa]	mSigma	rdb	e⁻ Conf	N-Rule
233.0806	1	C13H13O4	100.00	233.0808	1.2	0.3	23.3	7.5	even	ok

wanjianjiao_LH-H1_neg_41_01_7090.d

Bruker Compass DataAnalysis 4.1 printed: 8/14/2019 3:04:36 PM by: SCSIO Page 1 of 1

Figure S8. HRESI-MS spectrum of the new compound **2**

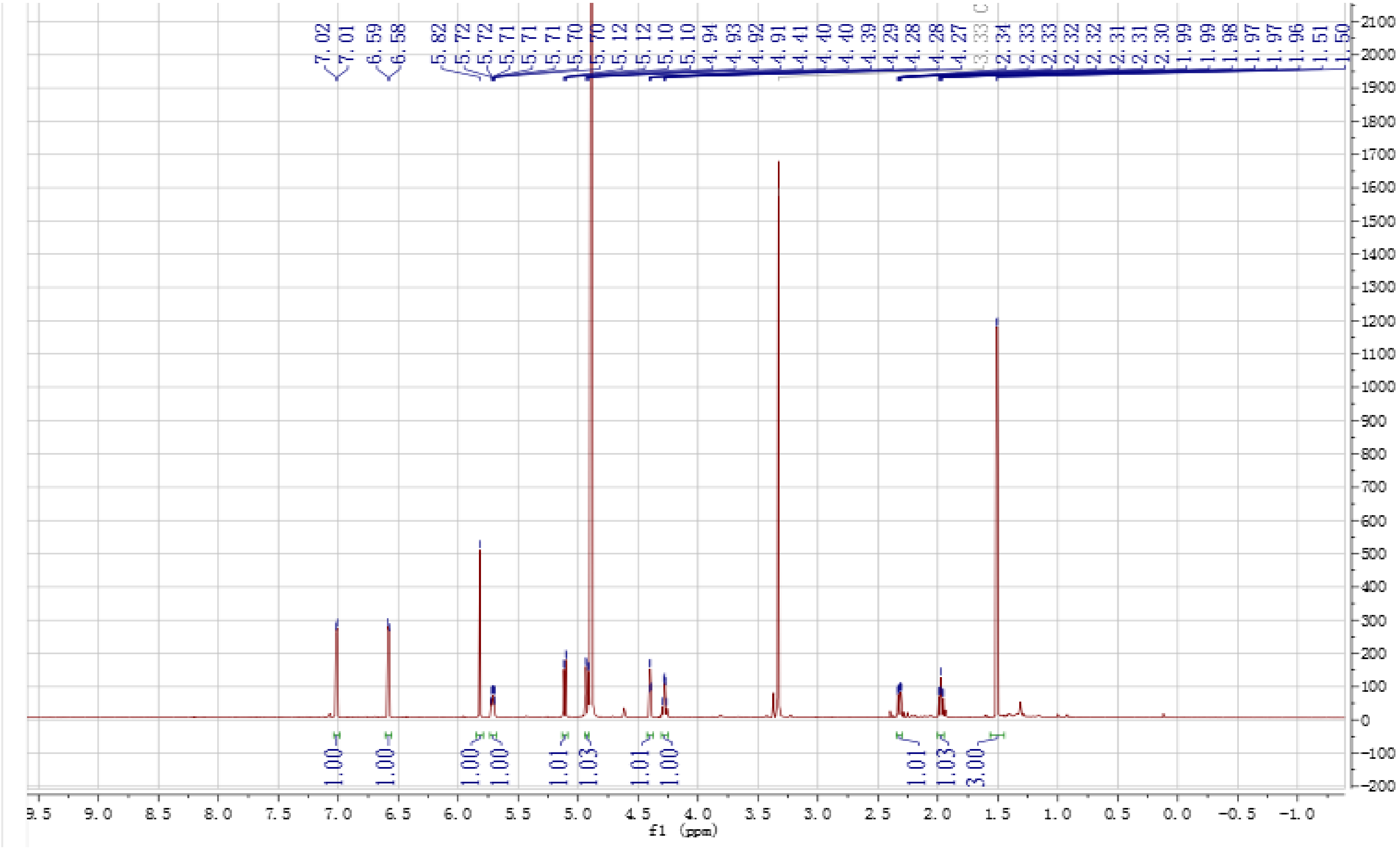

Figure S9. ^{1}H NMR (500 MHz, CD_3OD) spectrum of the new compound **2**

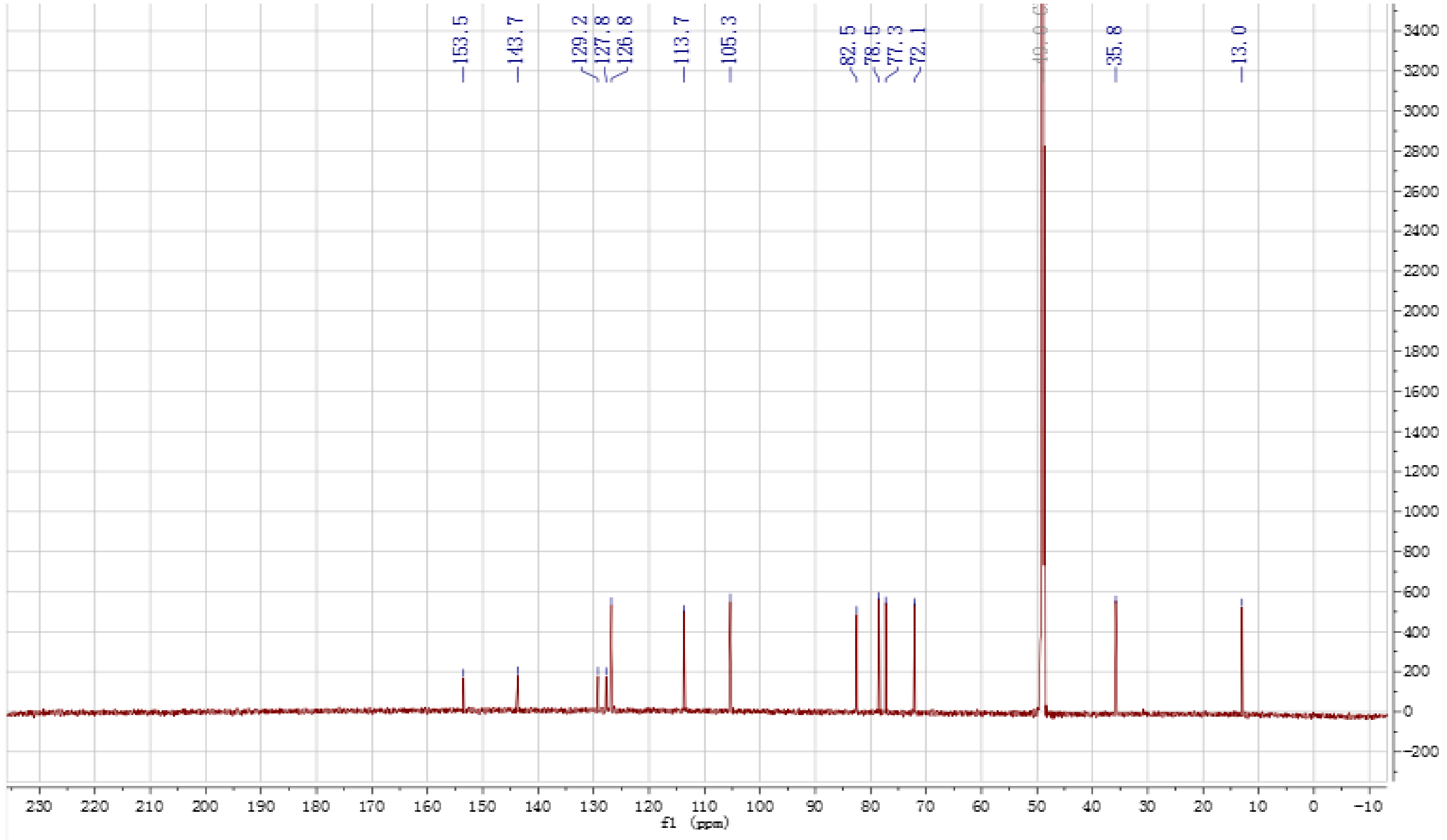

Figure S10. ^{13}C NMR (125 MHz, CD_3OD) spectrum of the newcompound **2**

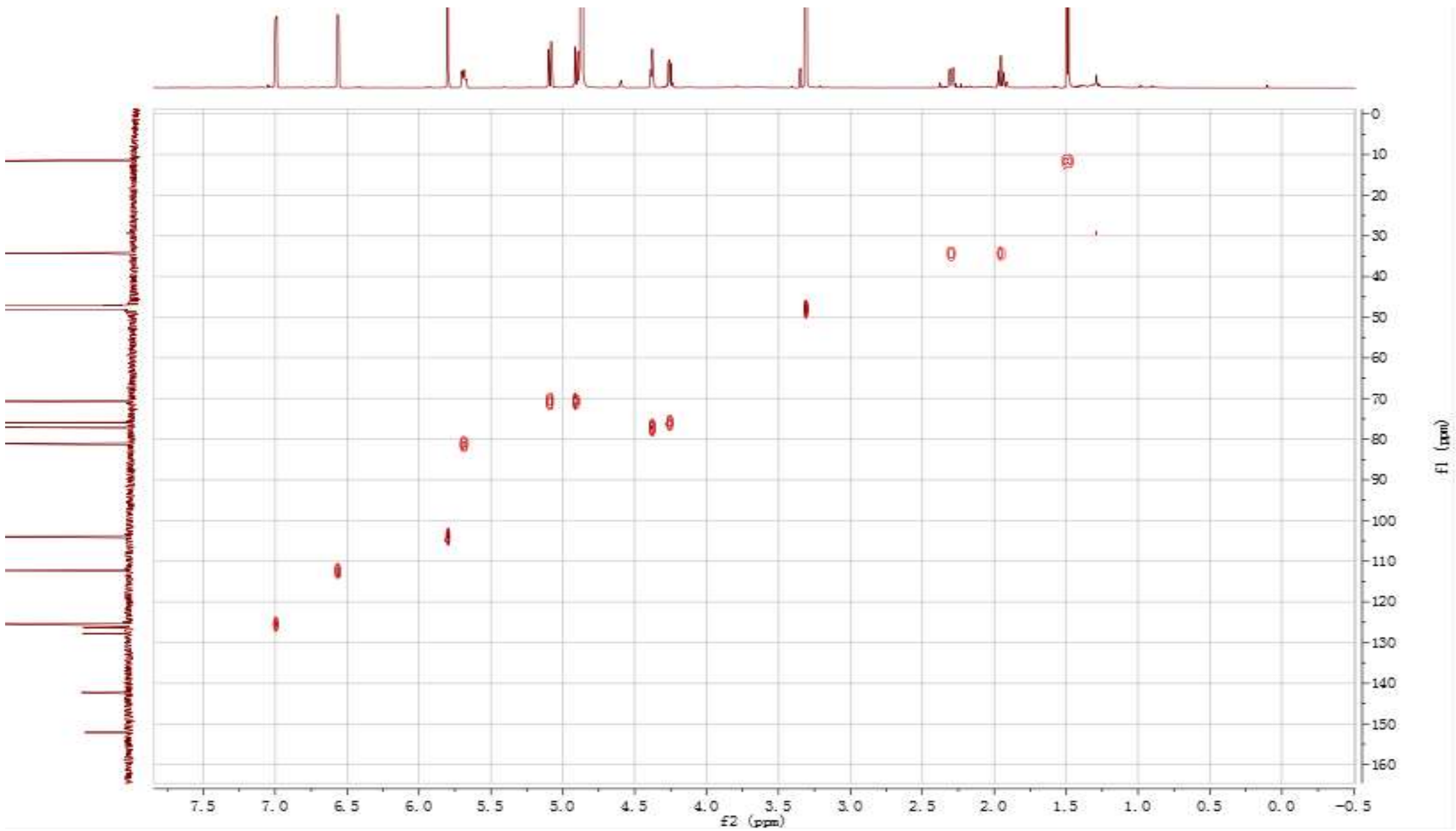

Figure S11. HSQC spectrum of the new compound **2**

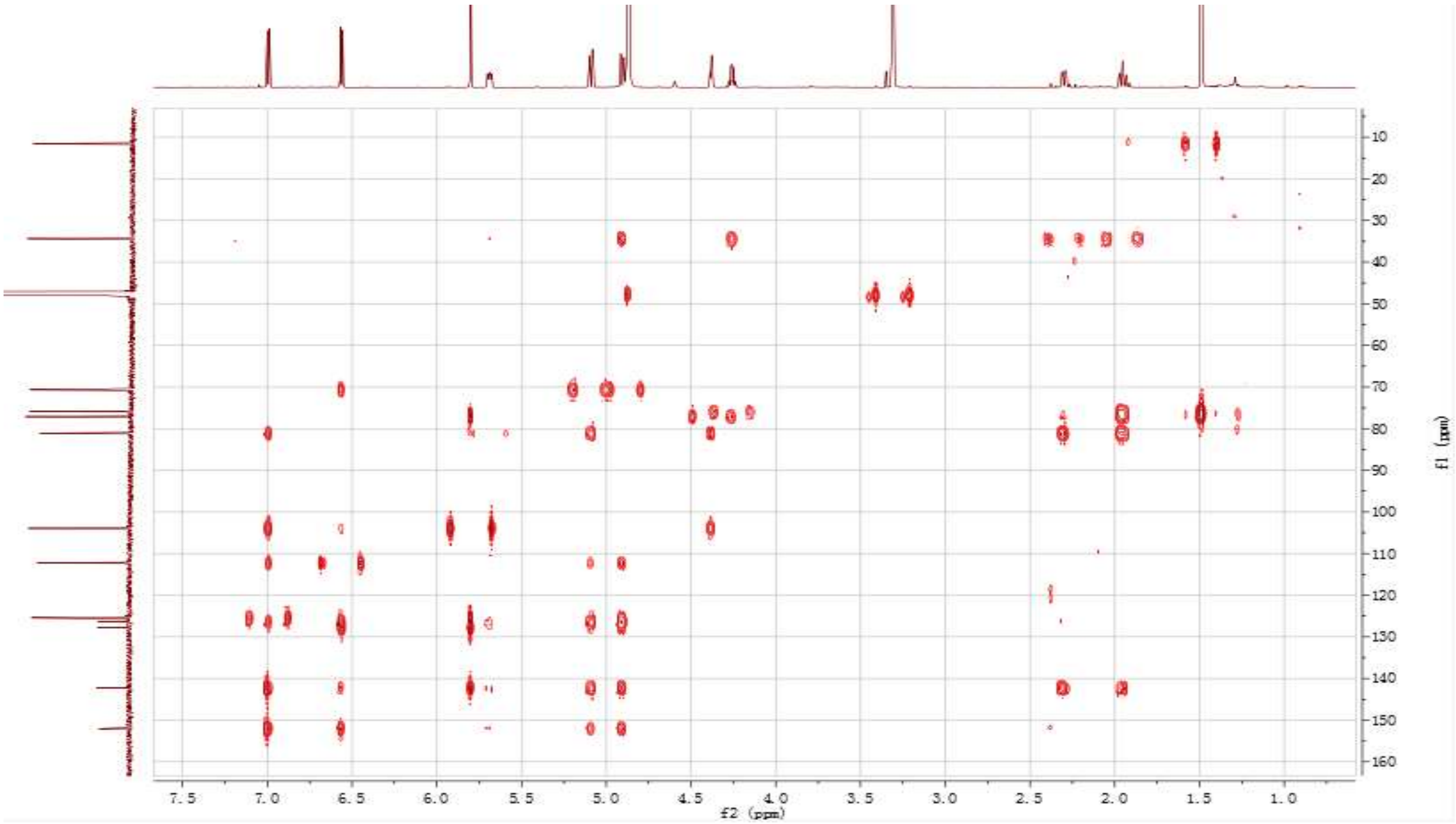

Figure S12. HMBC spectrum of the new compound **2**

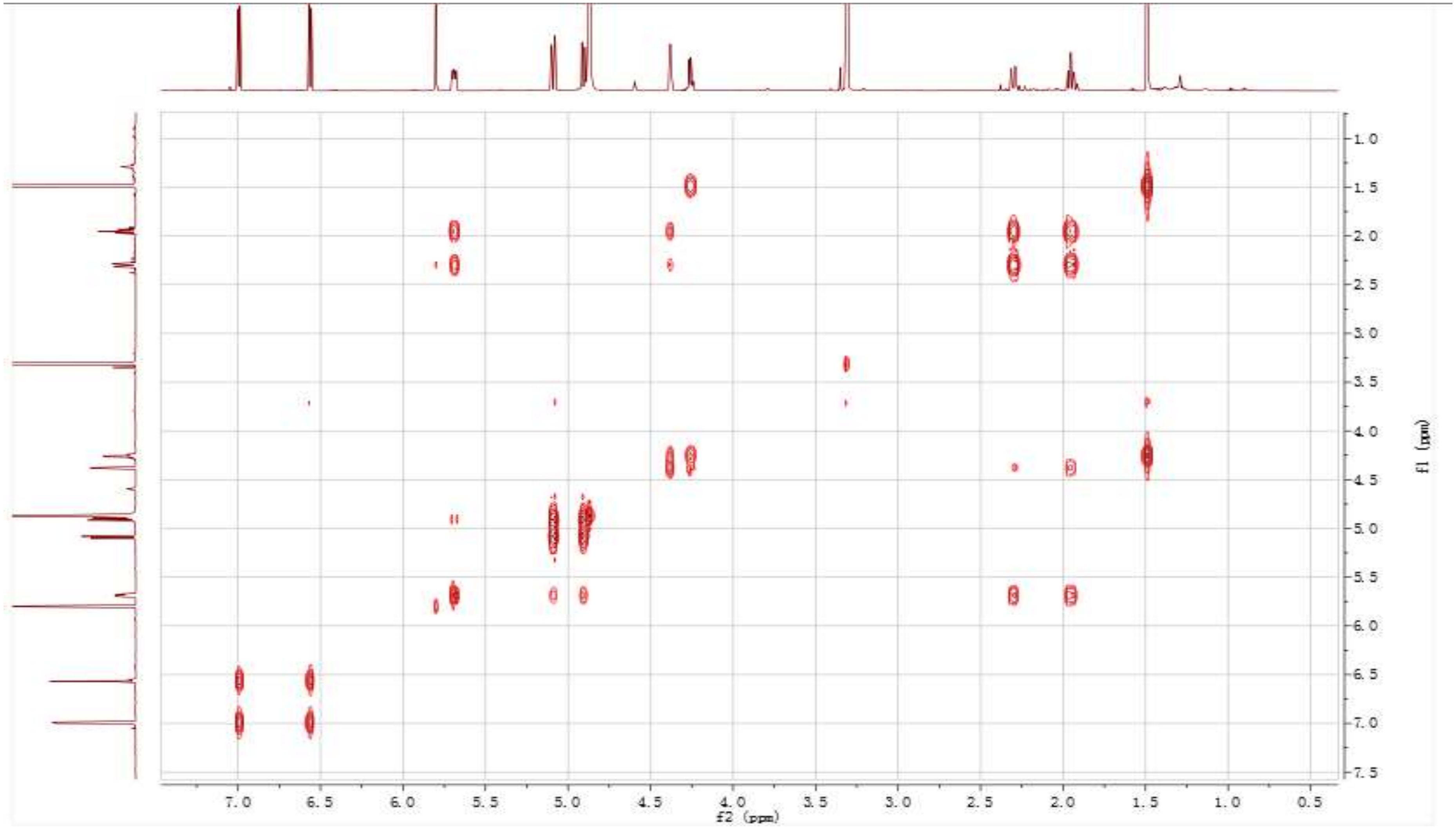

Figure S13. COSY spectrum of the new compound **2**

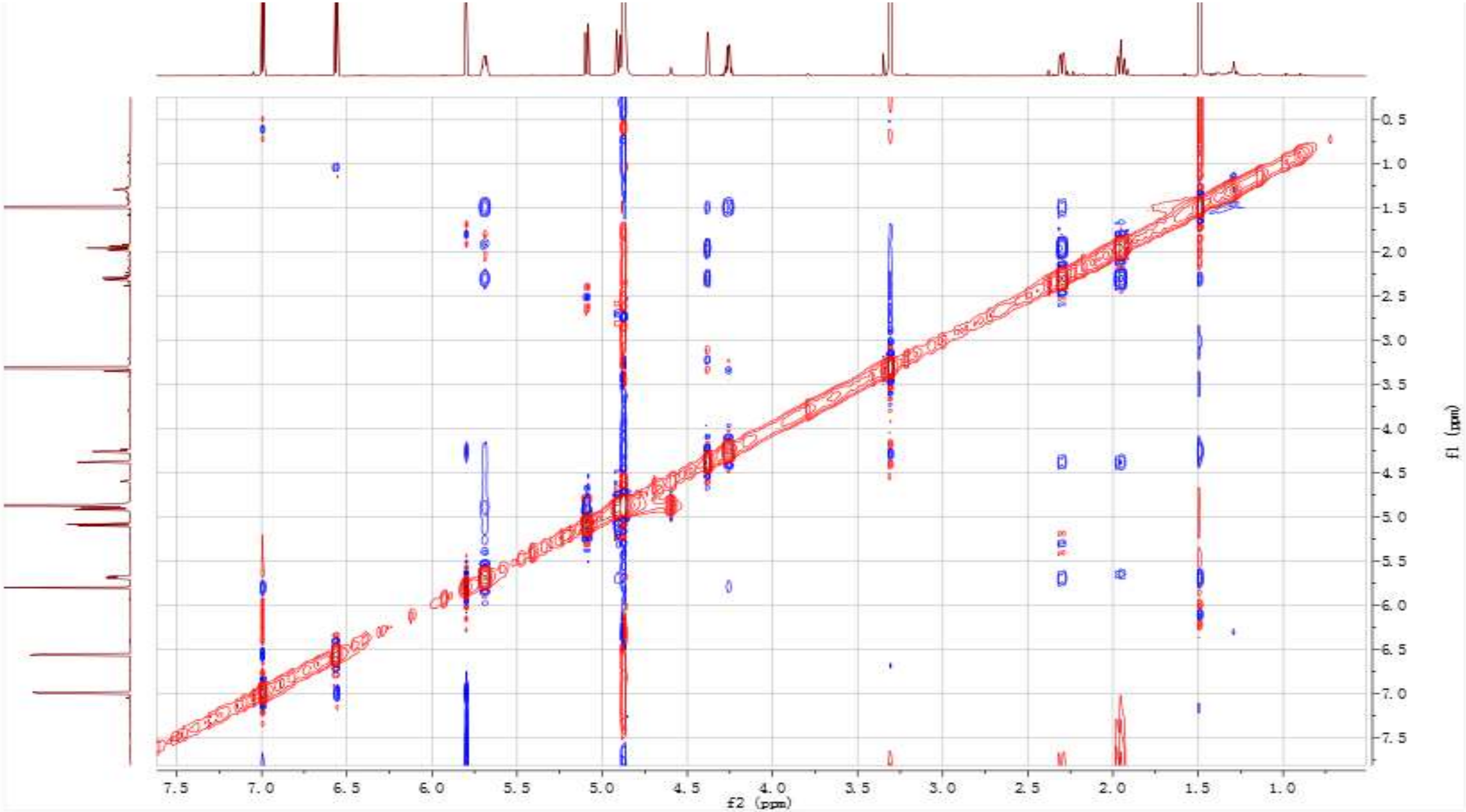

Figure S14. NOESY spectrum of the new compound **2**

Mass Spectrum SmartFormula Report

Analysis Info

Analysis Name	D:\Data\MS\data\201812\yangbin_L37_pos_22_01_5867.d	Acquisition Date	12/5/2018 3:53:15 PM
Method	LC_Direct Infusion_pos_70-500mz.m	Operator	SCSIO
Sample Name	yangbin_L37_pos	Instrument	maXis 255552.00029
Comment			

Acquisition Parameter

Source Type	ESI	Ion Polarity	Positive	Set Nebulizer	0.4 Bar
Focus	Active	Set Capillary	4500 V	Set Dry Heater	180 °C
Scan Begin	70 m/z	Set End Plate Offset	-500 V	Set Dry Gas	4.0 l/min
Scan End	1500 m/z	Set Charging Voltage	0 V	Set Divert Valve	Waste
		Set Corona	0 nA	Set APCI Heater	0 °C

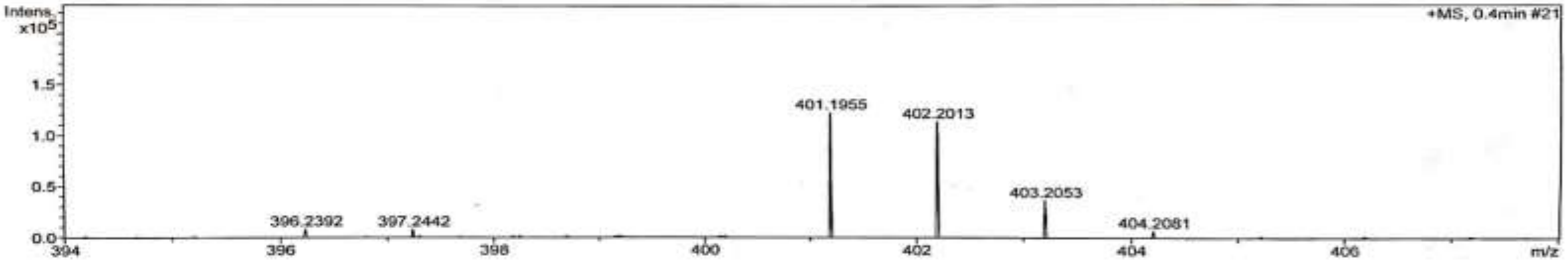

Meas. m/z	#	Ion Formula	Score	m/z	err [ppm]	err [mDa]	mSigma	rdb	e⁻ Conf	N-Rule
379.2100	1	C21H31O6	100.00	379.2115	-4.0	-1.5	291.1	6.5	even	ok
401.1955	1	C21H30NaO6	100.00	401.1935	5.2	2.1	372.7	6.5	even	ok
779.4017	1	C42H60NaO12	100.00	779.3977	5.2	4.0	483.3	12.5	even	ok

yangbin_L37_pos_22_01_5867.d
Bruker Compass DataAnalysis 4.1 printed: 12/5/2018 4:00:48 PM by: SCSIO Page 1 of 1

Figure S15. HRESI-MS spectrum of the new compound **3**

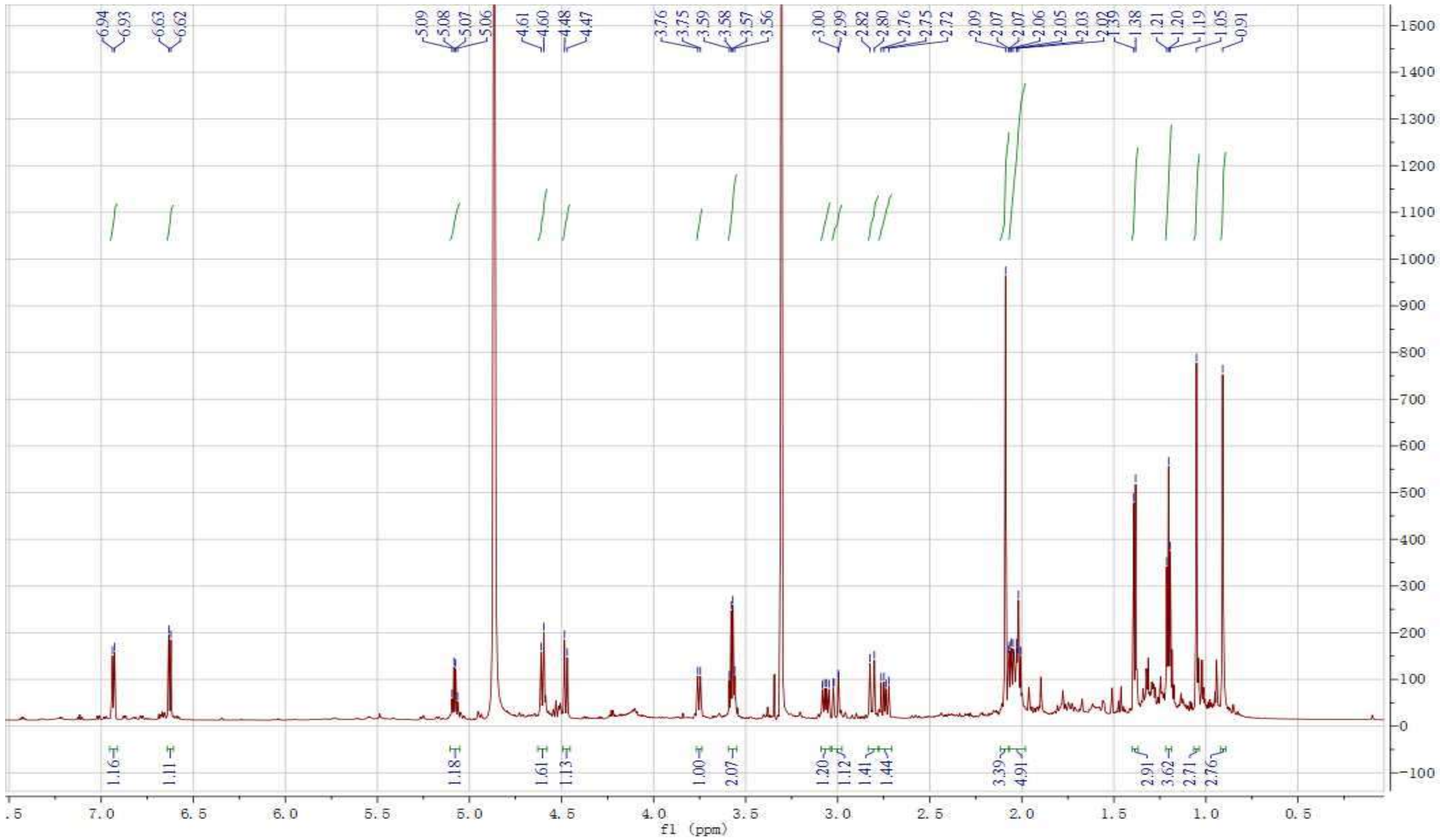

Figure S16. ^{1}H NMR (500 MHz, CD_3OD) spectrum of the new compound **3**

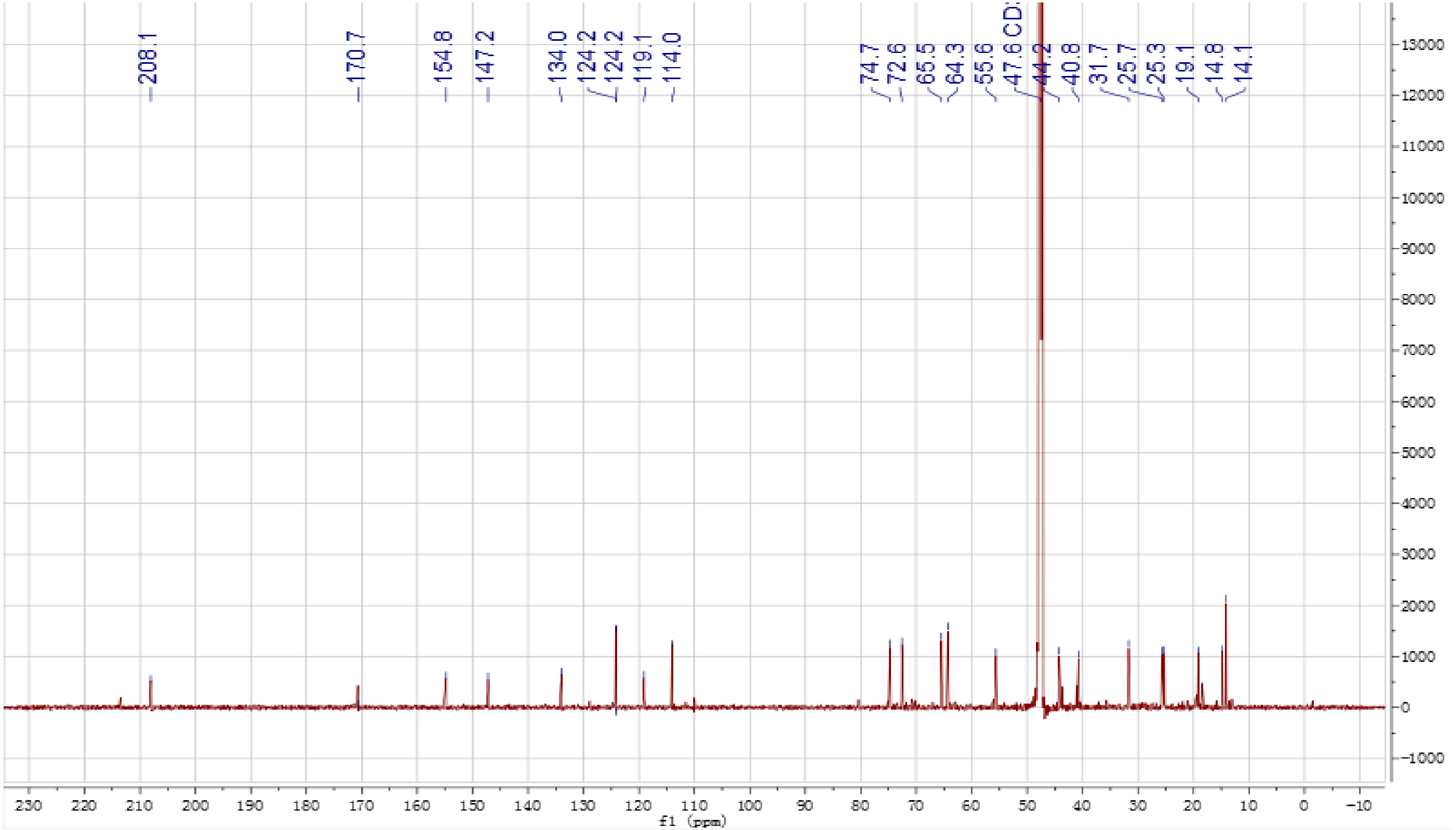

Fiure S17. ^{13}C NMR (125 MHz, CD_3OD) spectrum of the new compound **3**

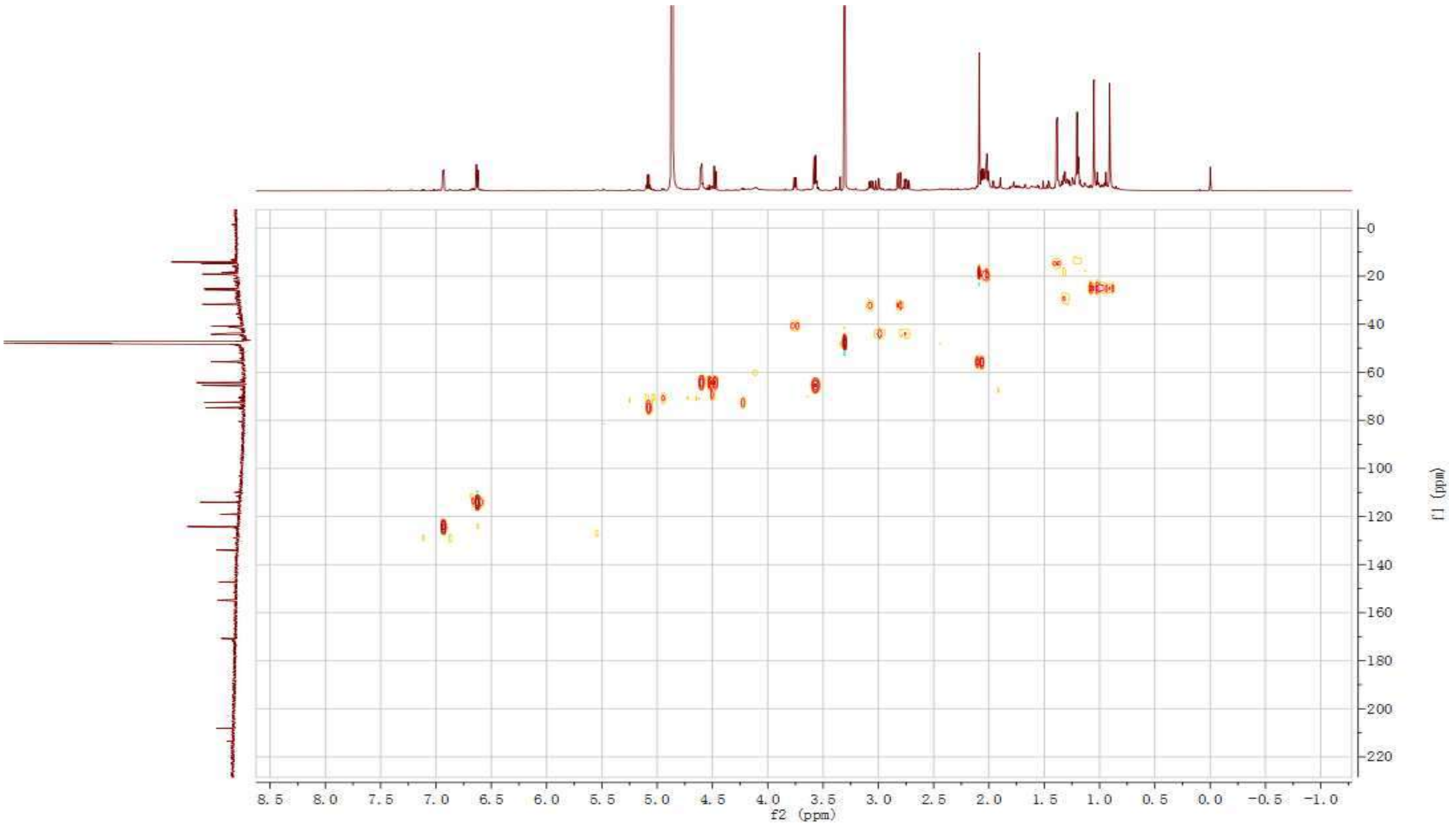

Figure S18. HSQC spectrum of the new compound **3**

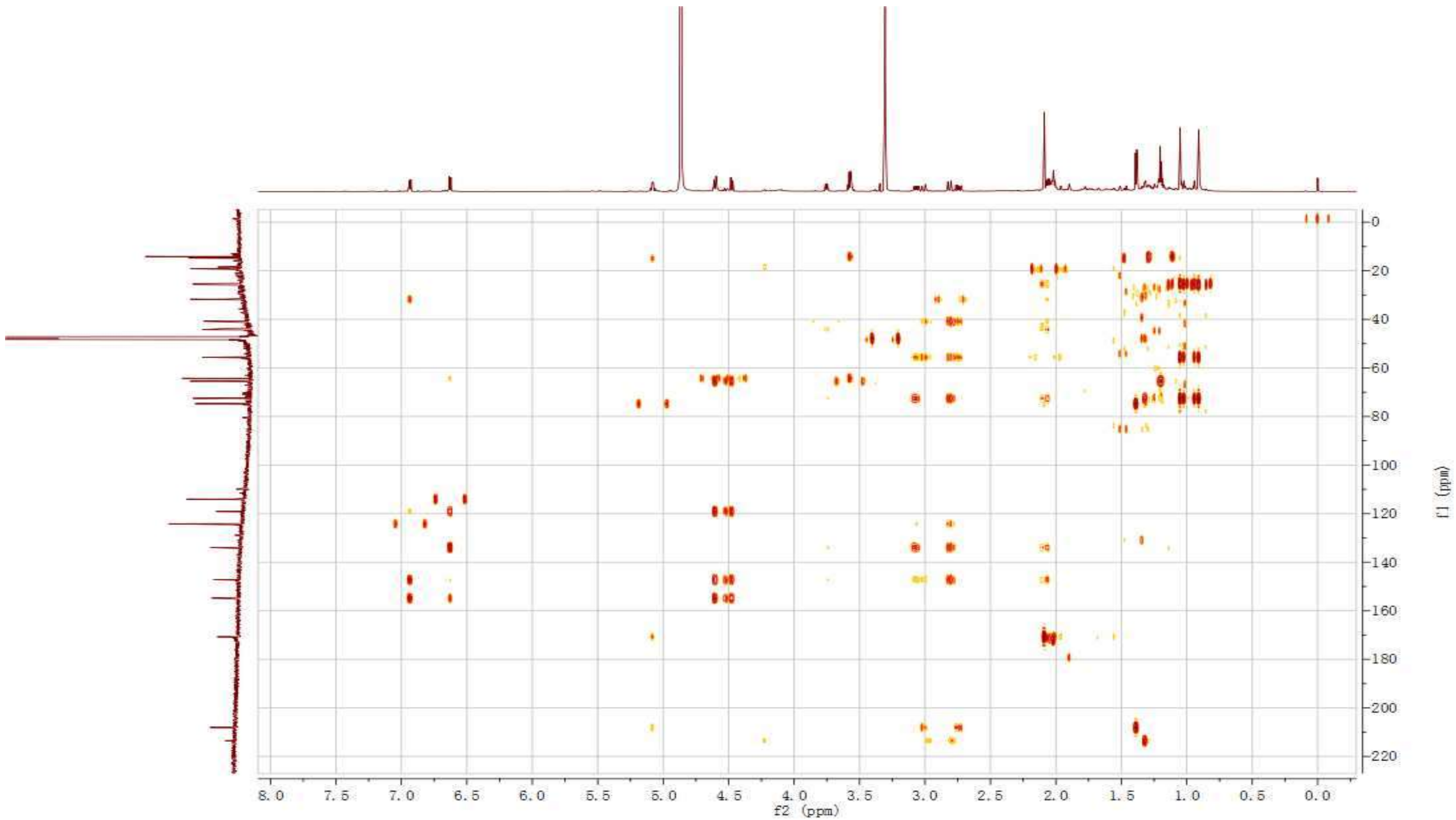

Figure S19. HMBC spectrum of the new compound **3**

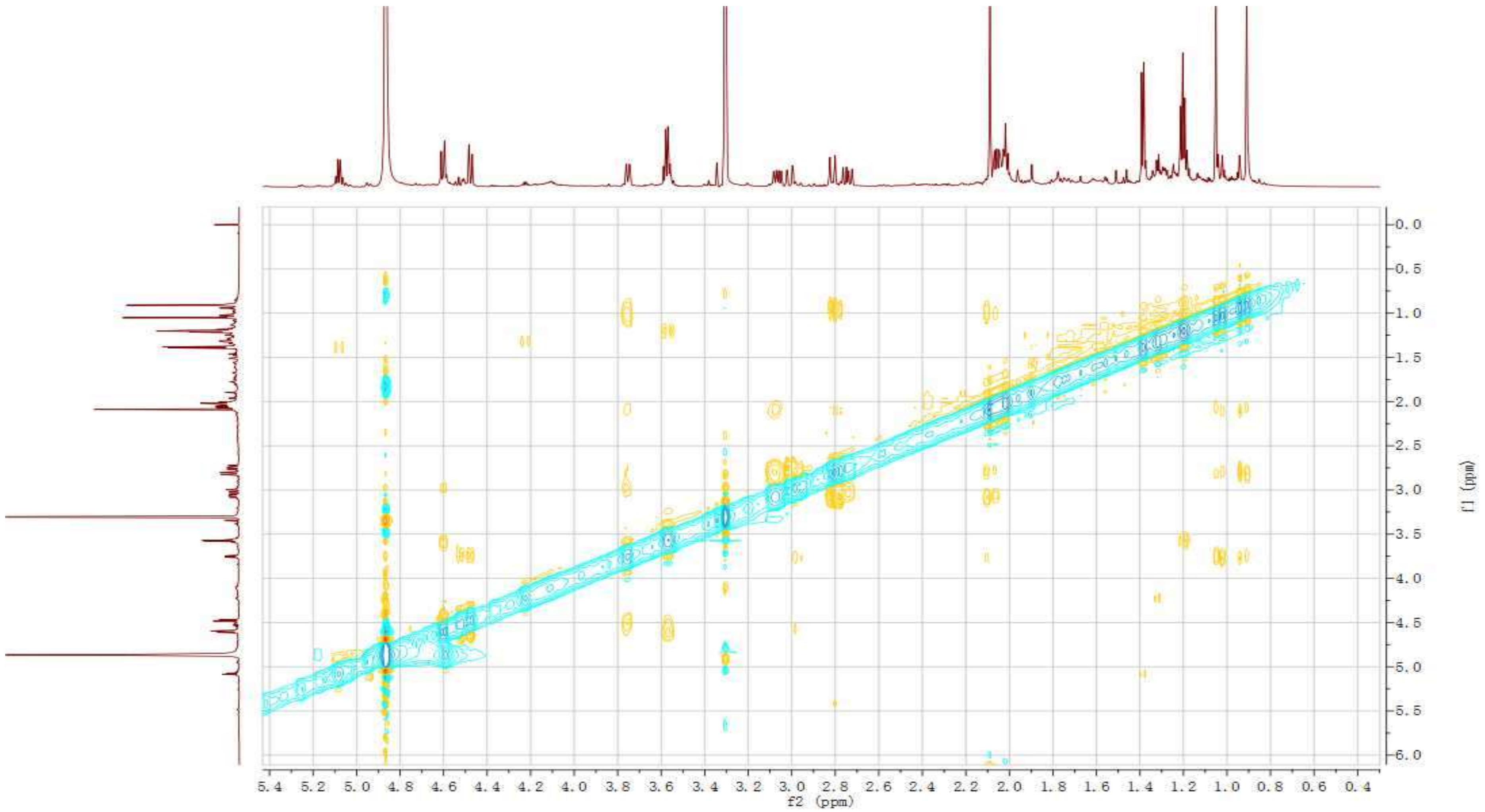

Figure S20. NOESY spectrum of the new compound **3**

Mass Spectrum SmartFormula Report

Analysis Info			
Analysis Name	D:\Data\MS\data\201812\yangbin_L38_pos_23_01_5868.d	Acquisition Date	12/5/2018 3:56:42 PM
Method	LC_Direct Infusion_pos_70-500mz.m	Operator	SCSIO
Sample Name	yangbin_L38_pos	Instrument	maXis 255552.00029
Comment			

Acquisition Parameter

Source Type	ESI	Ion Polarity	Positive	Set Nebulizer	0.4 Bar
Focus	Active	Set Capillary	4500 V	Set Dry Heater	180 °C
Scan Begin	70 m/z	Set End Plate Offset	-500 V	Set Dry Gas	4.0 l/min
Scan End	1500 m/z	Set Charging Voltage	0 V	Set Divert Valve	Waste
		Set Corona	0 nA	Set APCI Heater	0 °C

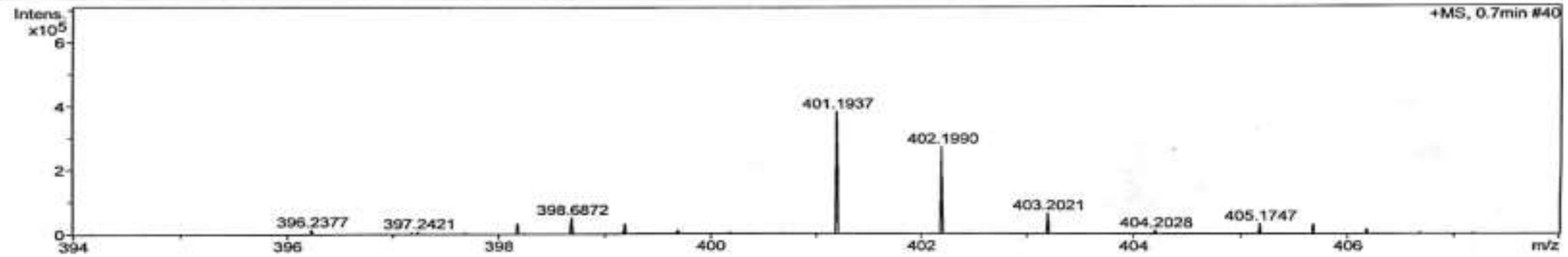

Meas. m/z	#	Ion Formula	Score	m/z	err [ppm]	err [mDa]	mSigma	rdb	e⁻ Conf	N-Rule
401.1937	1	C21H30NaO6	100.00	401.1935	0.7	0.3	249.3	6.5	even	ok
779.3962	1	C42H60NaO12	100.00	779.3977	-1.9	-1.5	370.1	12.5	even	ok

yangbin_L38_pos_23_01_5868.d
Bruker Compass DataAnalysis 4.1 printed: 12/5/2018 4:01:55 PM by: SCSIO Page 1 of 1

Figure S21. HRESI-MS spectrum of the new compound **4**

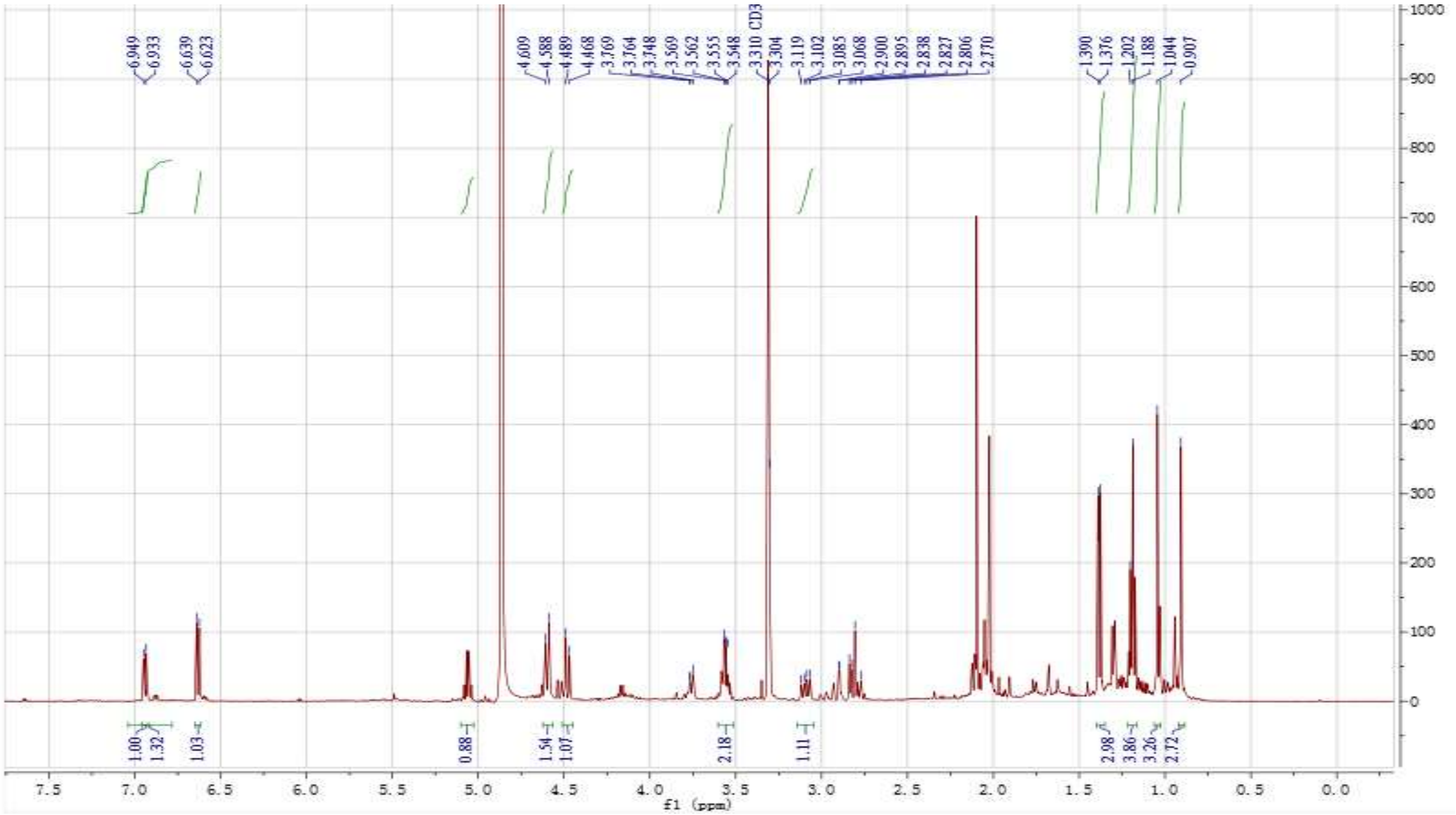

Figure S22. ^{1}H NMR (500 MHz, CD_3OD) spectrum of the new compound **4**

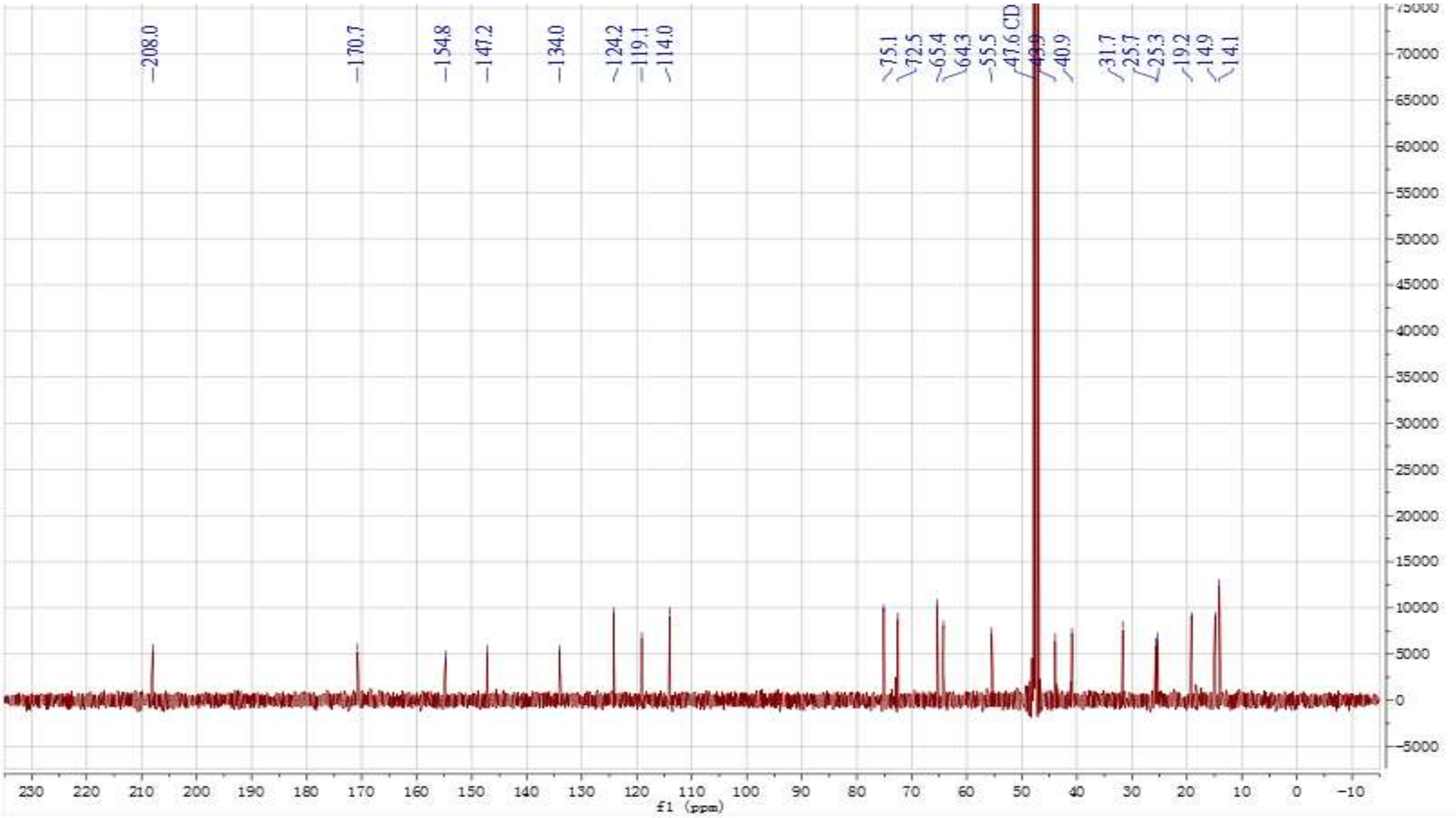

Figure S23. ^{13}C NMR (125 MHz, CD_3OD) spectrum of the new compound **4**

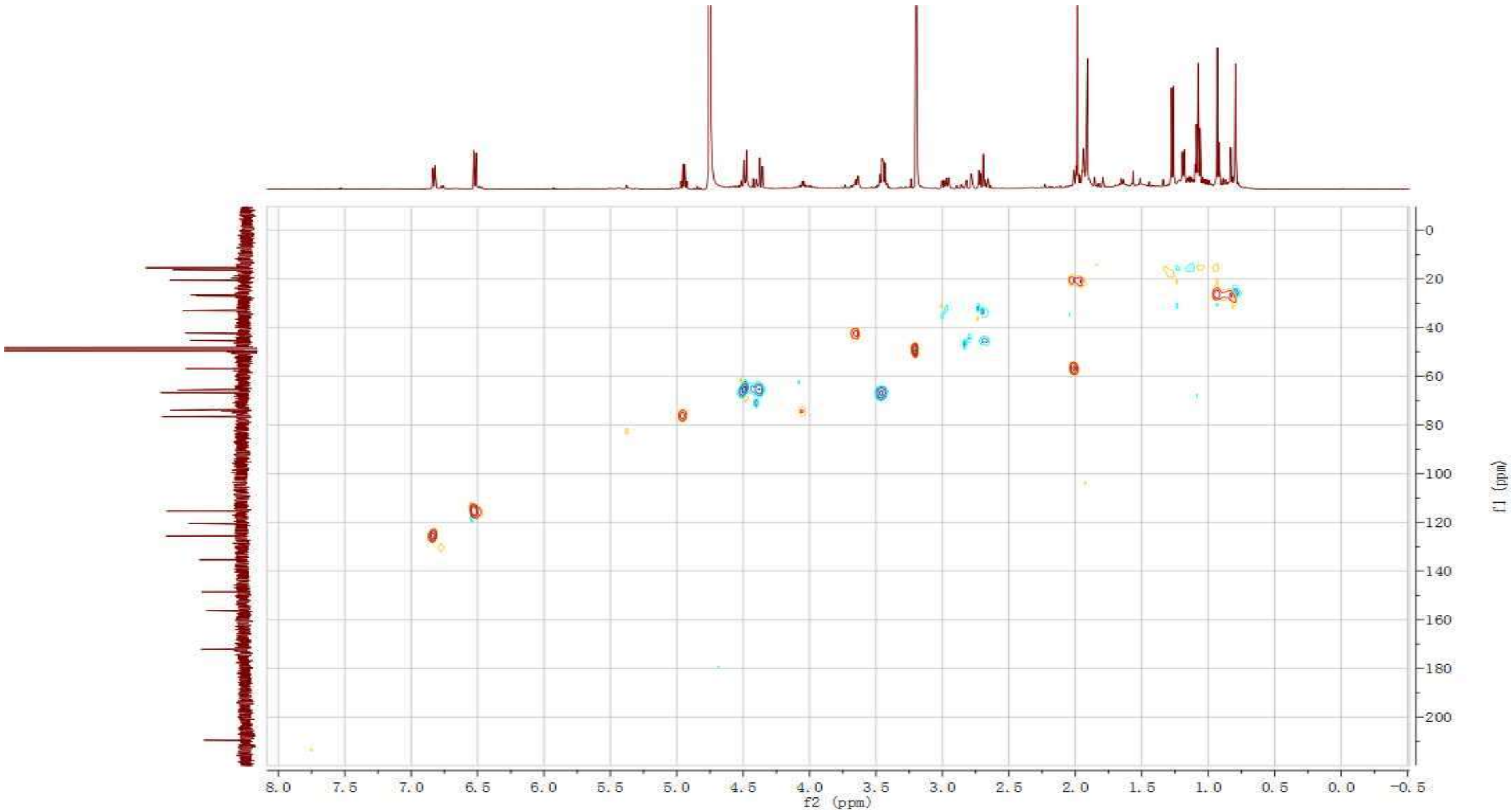

Figure S24. HSQC spectrum of the new compound **4**

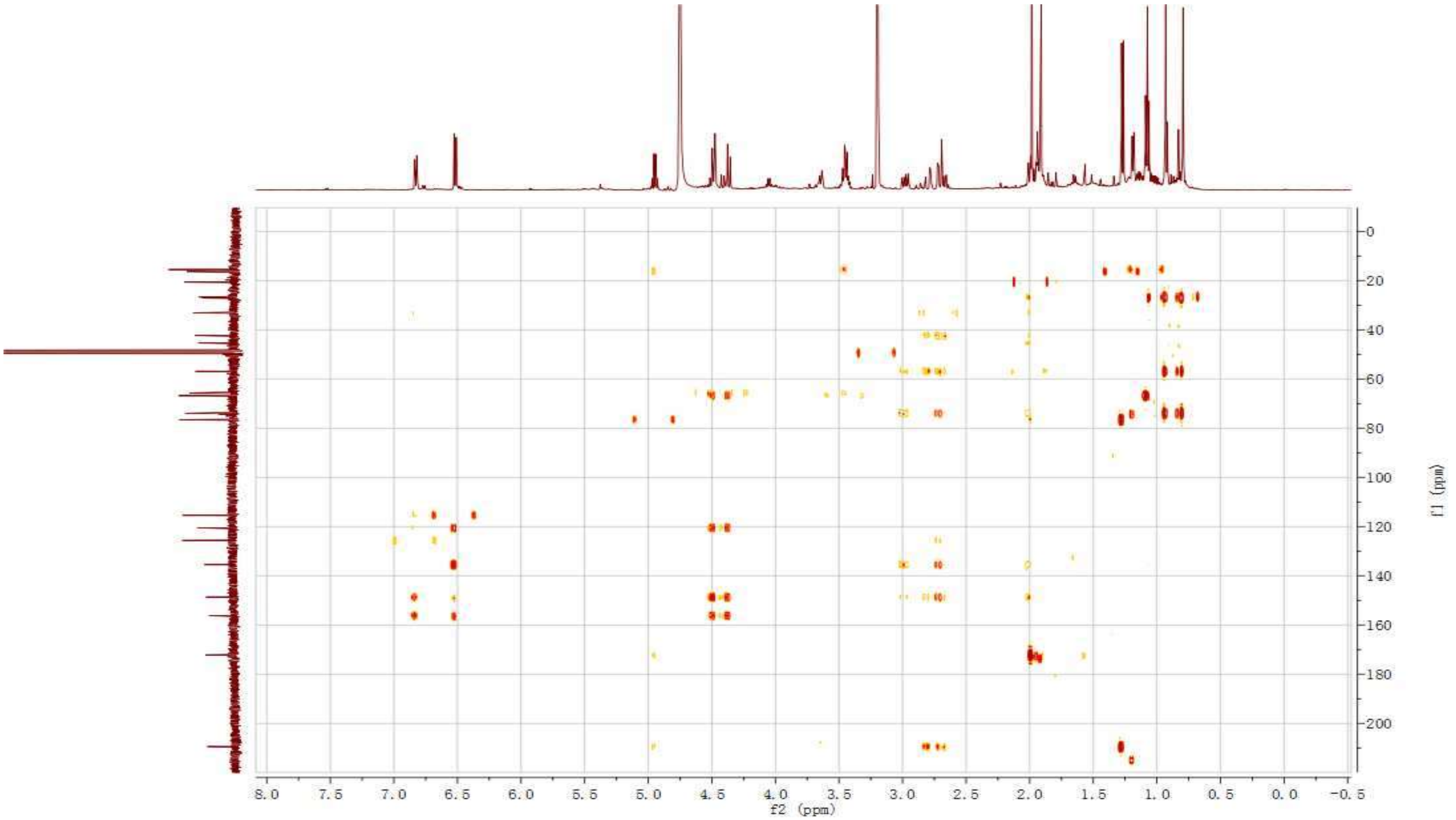

Figure S25. HMBC spectrum of the new compound **4**

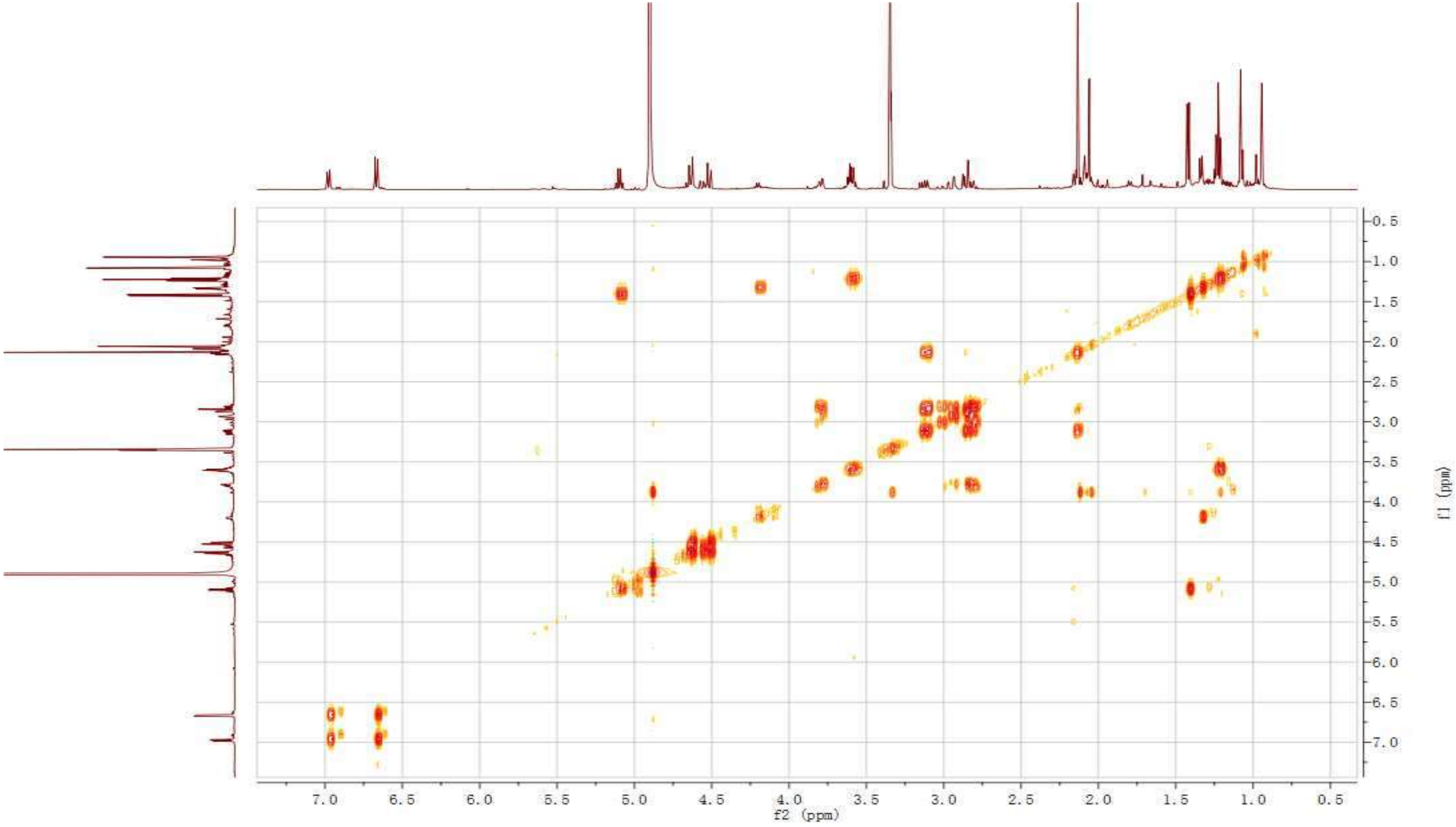

Figure S26. COSY spectrum of the new compound **4**

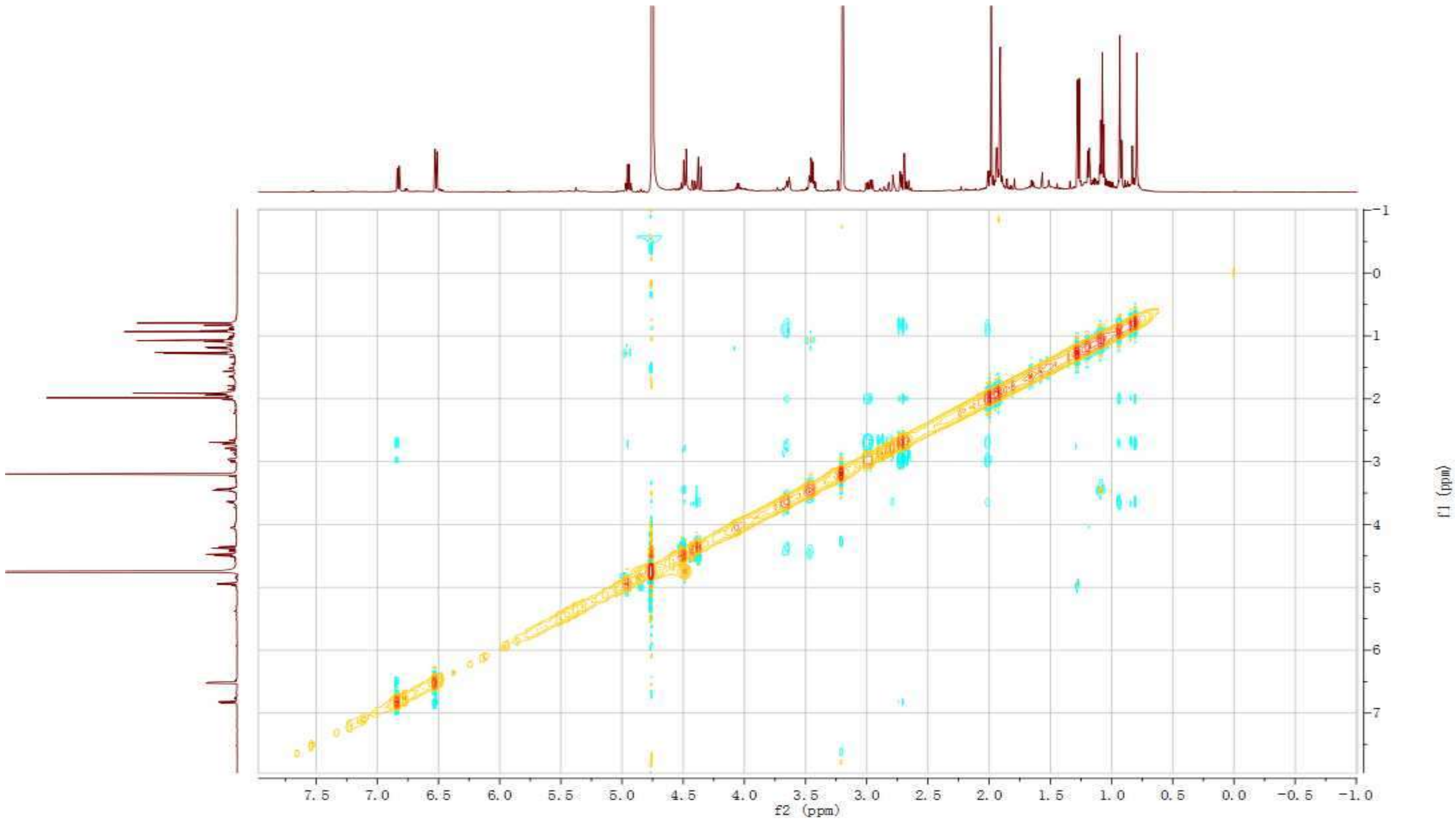

Figure S27. NOESY spectrum of the new compound **4**

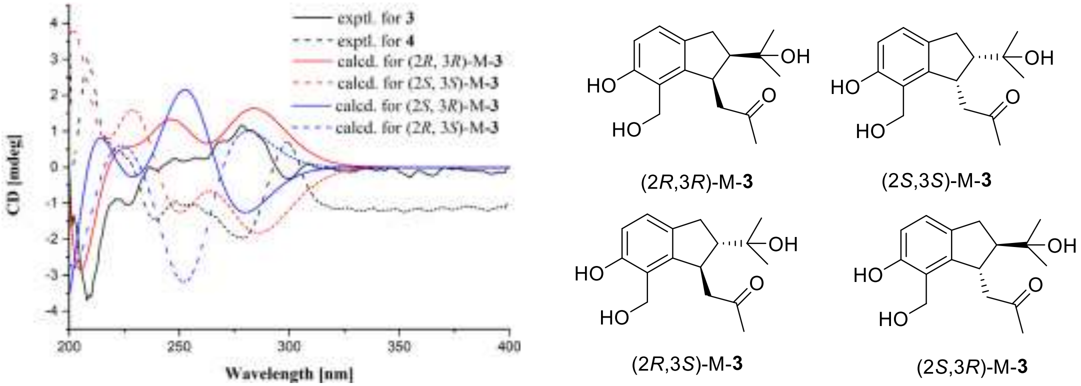

Figure S28. calculated and experimental ECD spectras of **3** and **4** using a truncated model.

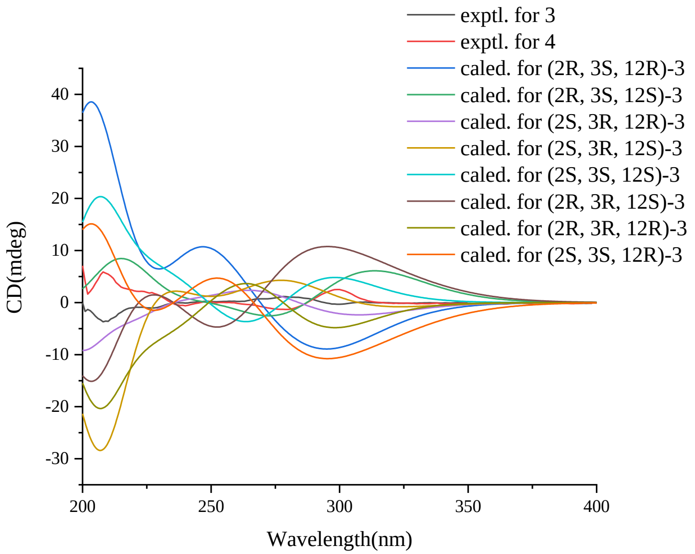

Figure S29. calculated and experimental ECD spectras of **3** and **4**.

Functional		Solvent?		Basis Set		Type of Data					
mPW1PW91		PCM		6-31G(d)		Shielding Tensors					
		DP4+	0.00%	0.00%	0.00%	0.00%	50.08%	25.45%	4.38%	#####	–
Nuclei	sp2?	xperimenta	Isomer 1	Isomer 2	Isomer 3	Isomer 4	Isomer 5	Isomer 6	Isomer 7	Isomer 8	Isomer 9
C	x	114	85.2	84.9	85.1	85.2	84.5	84.3	84.2	84.5	
C	x	154.8	44.4	44.4	44.4	44.4	45.1	44.6	44.6	45.2	
C	x	119.1	83.1	82.8	83.1	83.0	81.9	82.4	82.6	82.2	
C	x	147.4	50.7	50.7	50.5	50.7	52.8	52.9	53.0	52.8	
C	x	134	66.8	67.2	67.1	66.8	65.2	65.2	65.0	65.2	
C	x	124.2	73.8	73.9	73.9	73.8	73.9	73.7	73.7	74.0	
C		40.8	153.6	153.2	154.0	153.6	151.4	151.8	151.8	151.3	
C		55.6	141.8	142.2	142.2	141.9	141.9	140.3	140.3	141.7	
C		31.7	162.2	162.2	162.2	162.3	159.6	159.1	159.1	159.5	
C		64.3	128.3	127.7	127.9	128.1	128.2	127.3	127.5	128.3	
C		65.5	132.9	132.5	132.7	132.9	132.3	131.9	132.0	132.6	
C		14.1	179.19	178.85	179.16	179.17	178.9	178.5	178.5	179.0	
C		72.6	126.16	125.57	125.68	126.21	125.3	124.8	124.7	125.3	
C		25.3	162.93	163.07	163.08	163.00	171.7	170.8	170.8	171.8	
C		25.7	166.48	165.59	165.74	166.62	165.8	166.2	166.4	165.7	
C		44.2	152.79	154.80	154.57	152.79	148.9	148.2	147.6	148.8	
C	x	208.1	-4.73	-13.05	-10.65	-4.73	-6.8	-9.9	-9.8	-6.7	
C		74.7	117.68	120.33	119.12	117.78	119.4	118.5	118.7	119.4	
C	x	170.7	28.16	28.39	28.10	28.15	27.3	26.2	26.0	27.3	
C		14.8	178.44	176.35	176.40	178.34	178.0	177.8	177.7	177.9	
C		19.1	172.97	172.68	172.74	173.03	173.0	172.3	172.0	173.0	

Figure S30. The data of DP4plus method of compound **3**

Functional	Solvent?		Basis Set		Type of Data				
mPW1PW91	PCM		6-31G(d)		Shielding Tensors				
	Isomer 1	Isomer 2	Isomer 3	Isomer 4	Isomer 5	Isomer 6	Isomer 7	Isomer 8	Isomer 9
sDP4+ (H data)	–	–	–	–	–	–	–	–	–
sDP4+ (C data)	0.42%	0.00%	0.00%	0.54%	58.90%	1.40%	0.61%	38.12%	–
sDP4+ (all data)	0.42%	0.00%	0.00%	0.54%	58.90%	1.40%	0.61%	38.12%	–
uDP4+ (H data)	–	–	–	–	–	–	–	–	–
uDP4+ (C data)	0.01%	0.00%	0.01%	0.02%	3.17%	68.04%	26.79%	1.97%	–
uDP4+ (all data)	0.01%	0.00%	0.01%	0.02%	3.17%	68.04%	26.79%	1.97%	–
DP4+ (H data)	–	–	–	–	–	–	–	–	–
DP4+ (C data)	0.00%	0.00%	0.00%	0.00%	50.08%	25.45%	4.38%	20.08%	–
DP4+ (all data)	0.00%	0.00%	0.00%	0.00%	50.08%	25.45%	4.38%	20.08%	–

Figure S31. Comparison of the experimental ^{13}C NMR data and mean absolute error of compound **3**

configuration	*R2*	RMSE	DP4+(%)
**a***(2R,3R,12R)*	0.9982	2.52	0.00
**b***(2R,3R,12S)*	0.9973	3.16	0.00
**c***(2S,3S,12R)*	0.9977	2.91	0.00
**d***(2S,3S,12S)*	0.9982	2.51	0.00
**e***(2R,3S,12R)*	0.9987	2.20	50.08
**f***(2S,3R,12R)*	0.9985	2.65	25.45
**g***(2R,3S,12S)*	0.9984	2.70	4.38
**h***(2S,3R,12S)*	0.9987	2.22	20.08

Figure S32. $DP4^+$ probability of ^{13}C NMR chemical shifts（compound **3**）.

	A	B	C	D	E	F	G	H	I	J	K	L
2	mPW1PW91		PCM		6-31G(d)		Shielding Tensors					
3												
12			DP4+	0.00%	0.00%	0.00%	0.00%	51.62%	23.60%	4.23%	#####	–
14	Nuclei	sp2?	xperimenta	Isomer 1	Isomer 2	Isomer 3	Isomer 4	Isomer 5	Isomer 6	Isomer 7	Isomer 8	Isomer 9
15	C	x	114	85.2	84.9	85.1	85.2	84.5	84.3	84.2	84.5	
16	C	x	154.8	44.4	44.4	44.4	44.4	45.1	44.6	44.6	45.2	
17	C	x	119.1	83.1	82.8	83.1	83.0	81.9	82.4	82.6	82.2	
18	C	x	147.2	50.7	50.7	50.5	50.7	52.8	52.9	53.0	52.8	
19	C	x	134	66.8	67.2	67.1	66.8	65.2	65.2	65.0	65.2	
20	C	x	124.2	73.8	73.9	73.9	73.8	73.9	73.7	73.7	74.0	
21	C		40.9	153.6	153.2	154.0	153.6	151.4	151.8	151.8	151.3	
22	C		55.5	141.8	142.2	142.2	141.9	141.9	140.3	140.3	141.7	
23	C		31.7	162.2	162.2	162.2	162.3	159.6	159.1	159.1	159.5	
24	C		64.3	128.3	127.7	127.9	128.1	128.2	127.3	127.5	128.3	
25	C		65.4	132.9	132.5	132.7	132.9	132.3	131.9	132.0	132.6	
26	C		14.1	179.19	178.85	179.16	179.17	178.9	178.5	178.5	179.0	
27	C		72.5	126.16	125.57	125.68	126.21	125.3	124.8	124.7	125.3	
28	C		25.3	162.93	163.07	163.08	163.00	171.7	170.8	170.8	171.8	
29	C		25.7	166.48	165.59	165.74	166.62	165.8	166.2	166.4	165.7	
30	C		43.9	152.79	154.80	154.57	152.79	148.9	148.2	147.6	148.8	
31	C	x	208	-4.73	-13.05	-10.65	-4.73	-6.8	-9.9	-9.8	-6.7	
32	C		75.1	117.68	120.33	119.12	117.78	119.4	118.5	118.7	119.4	
33	C	x	170.7	28.16	28.39	28.10	28.15	27.3	26.2	26.0	27.3	
34	C		14.9	178.44	176.35	176.40	178.34	178.0	177.8	177.7	177.9	
35	C		19.2	172.97	172.68	172.74	173.03	173.0	172.3	172.0	173.0	
36												

Figure S33. The data of DP4plus method of compound **4**

Functional	Solvent?	Basis Set	Type of Data
mPW1PW91	PCM	6-31G(d)	Shielding Tensors

	Isomer 1	Isomer 2	Isomer 3	Isomer 4	Isomer 5	Isomer 6	Isomer 7	Isomer 8	Isomer 9
sDP4+ (H data)	–	–	–	–	–	–	–	–	–
sDP4+ (C data)	0.46%	0.00%	0.00%	0.59%	59.06%	1.22%	0.58%	38.09%	–
sDP4+ (all data)	0.46%	0.00%	0.00%	0.59%	59.06%	1.22%	0.58%	38.09%	–
uDP4+ (H data)	–	–	–	–	–	–	–	–	–
uDP4+ (C data)	0.02%	0.00%	0.01%	0.03%	3.12%	68.93%	25.97%	1.92%	–
uDP4+ (all data)	0.02%	0.00%	0.01%	0.03%	3.12%	68.93%	25.97%	1.92%	–
DP4+ (H data)	–	–	–	–	–	–	–	–	–
DP4+ (C data)	0.00%	0.00%	0.00%	0.00%	51.62%	23.60%	4.23%	20.53%	–
DP4+ (all data)	0.00%	0.00%	0.00%	0.00%	51.62%	23.60%	4.23%	20.53%	–

Figure S34. Comparison of the experimental ^{13}C NMR data and mean absolute error of compound **4**

configuration	R_2	RMSE	DP4+(%)
**a***(2R,3R,12R)*	0.9983	2.47	0.00
**b***(2R,3R,12S)*	0.9974	3.13	0.00
**c***(2S,3S,12R)*	0.9977	2.87	0.00
**d***(2S,3S,12S)*	0.9983	2.45	0.00
**e***(2R,3S,12R)*	0.9988	2.17	51.62
**f***(2S,3R,12R)*	0.9985	2.64	23.60
**g***(2R,3S,12S)*	0.9985	2.69	4.23
**h***(2S,3R,12S)*	0.9987	2.20	20.53

Figure S35. $DP4^+$ probability of ^{13}C NMR chemical shifts.

Mass Spectrum SmartFormula Report

Analysis Info

Analysis Name	D:\Data\MS\data\201907\wangjianjiao_LH-C1_pos_65_01_6940.d	Acquisition Date	7/16/2019 3:12:22 PM
Method	LC_Direct Infusion_pos_70-500mz.m	Operator	SCSIO
Sample Name	wangjianjiao_LH-C1_pos	Instrument	maXis 255552.00029
Comment			

Acquisition Parameter

Source Type	ESI	Ion Polarity	Positive	Set Nebulizer	0.4 Bar
Focus	Active	Set Capillary	4500 V	Set Dry Heater	180 °C
Scan Begin	70 m/z	Set End Plate Offset	-500 V	Set Dry Gas	4.0 l/min
Scan End	1500 m/z	Set Charging Voltage	0 V	Set Divert Valve	Waste
		Set Corona	0 nA	Set APCI Heater	0 °C

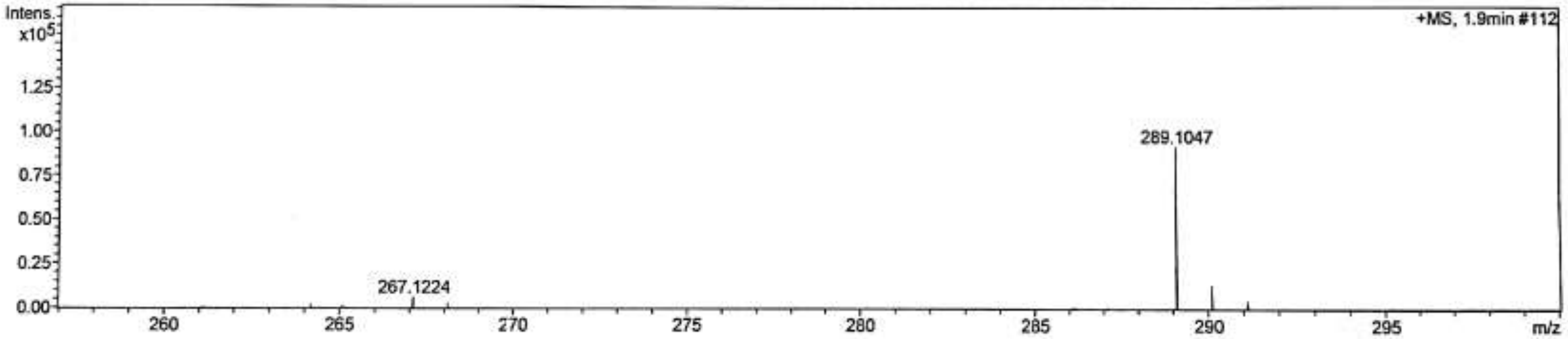

Meas. m/z	#	Ion Formula	Score	m/z	err [ppm]	err [mDa]	mSigma	rdb	e⁻ Conf	N-Rule
267.1224	1	C14H19O5	100.00	267.1227	1.3	0.3	15.4	5.5	even	ok
289.1047	1	C14H18NaO5	100.00	289.1046	-0.3	-0.1	10.1	5.5	even	ok
555.2195	1	C28H36NaO10	100.00	555.2201	1.0	0.6	28.8	10.5	even	ok

Figure S36. HRESI-MS spectrum of the new compound **5**

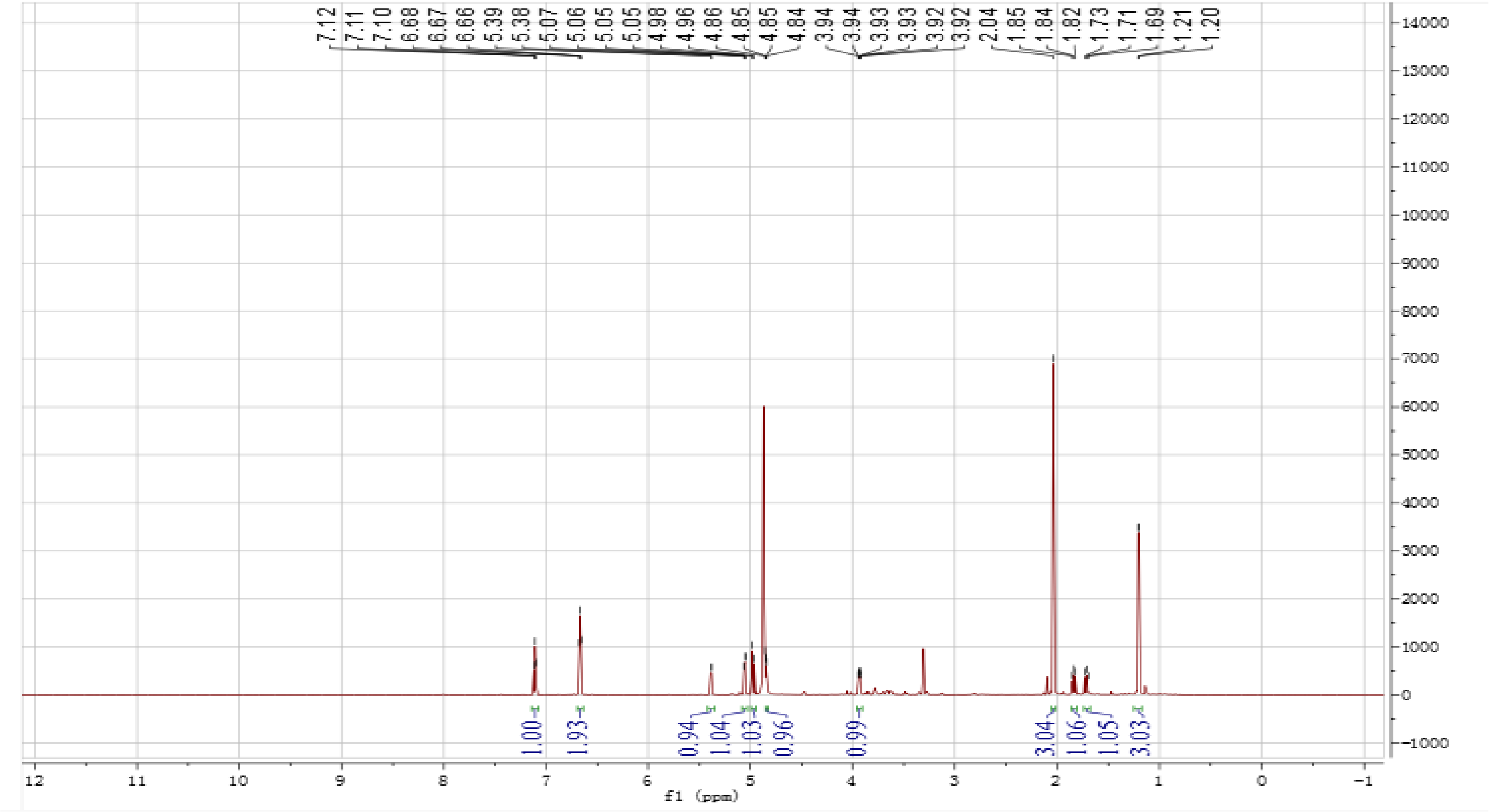

Figure S37. ^{1}H NMR (500 MHz, CD_3OD) spectrum of the new compound **5**

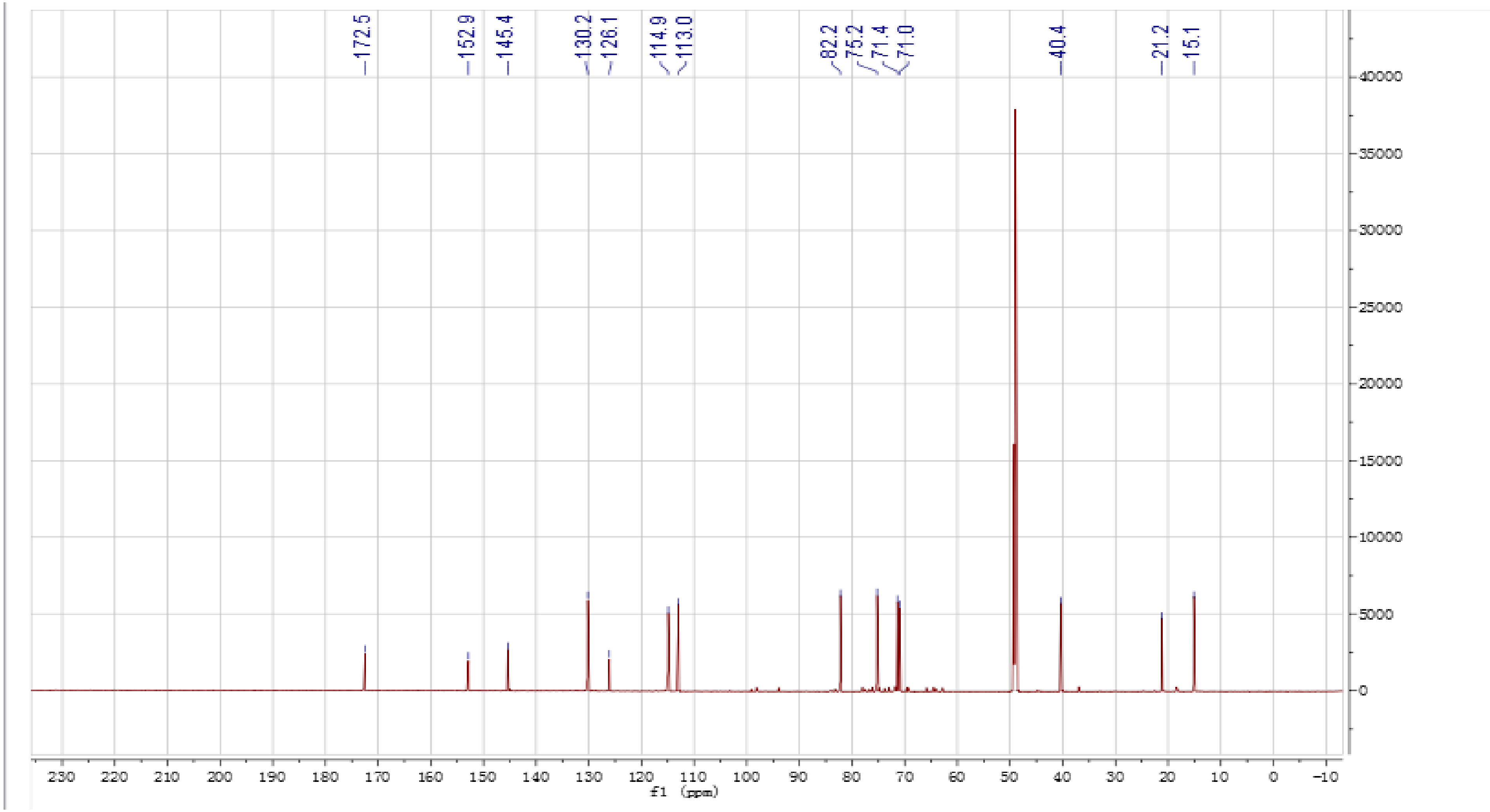

Figure S38. ^{13}C NMR (125 MHz, CD_3OD) spectrum of the new compound **5**

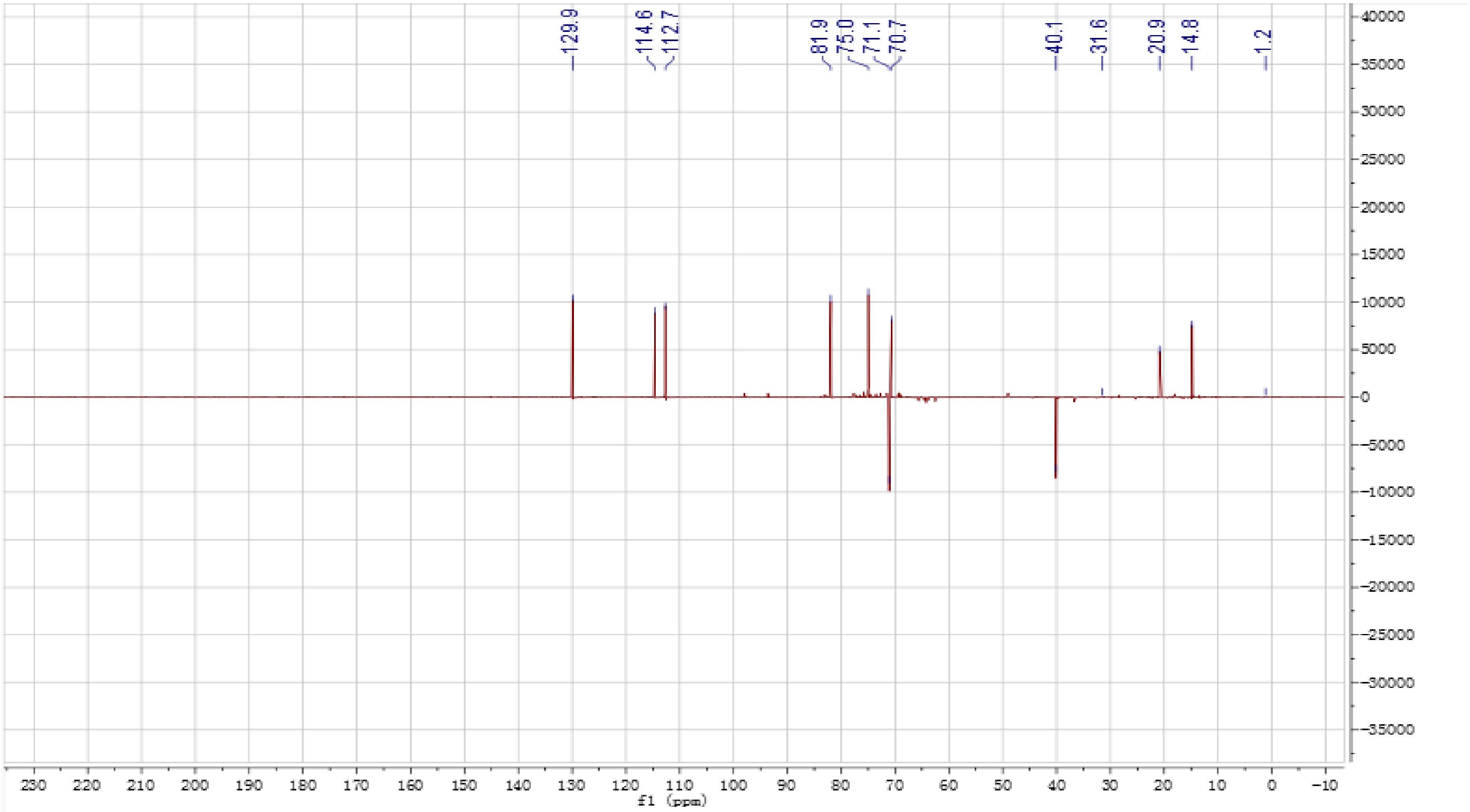

Figure S39. [135] DEPT (125 MHz, CD_3OD) spectrum of the new compound **5**

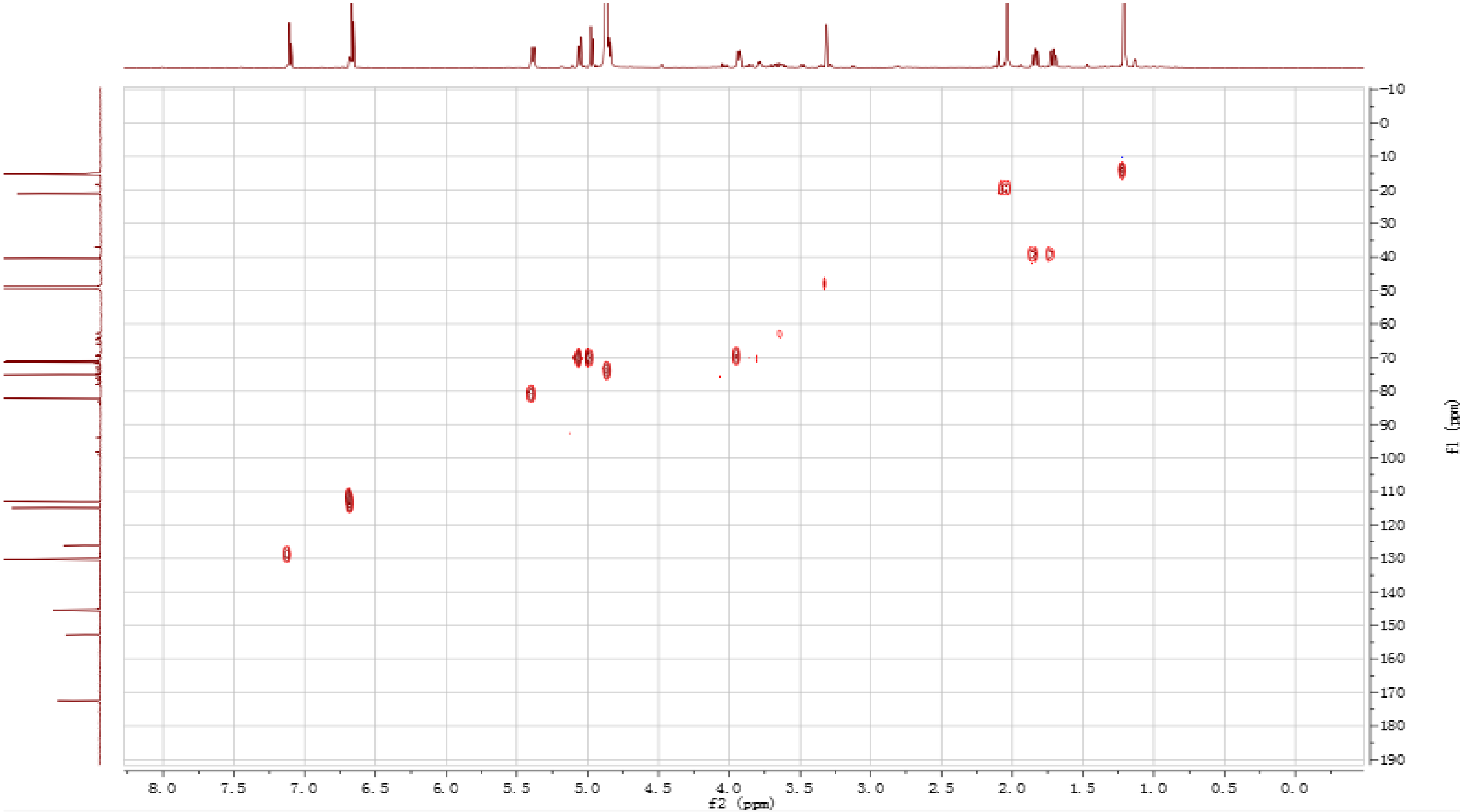

Figure S40. HSQC spectrum of the new compound **5**

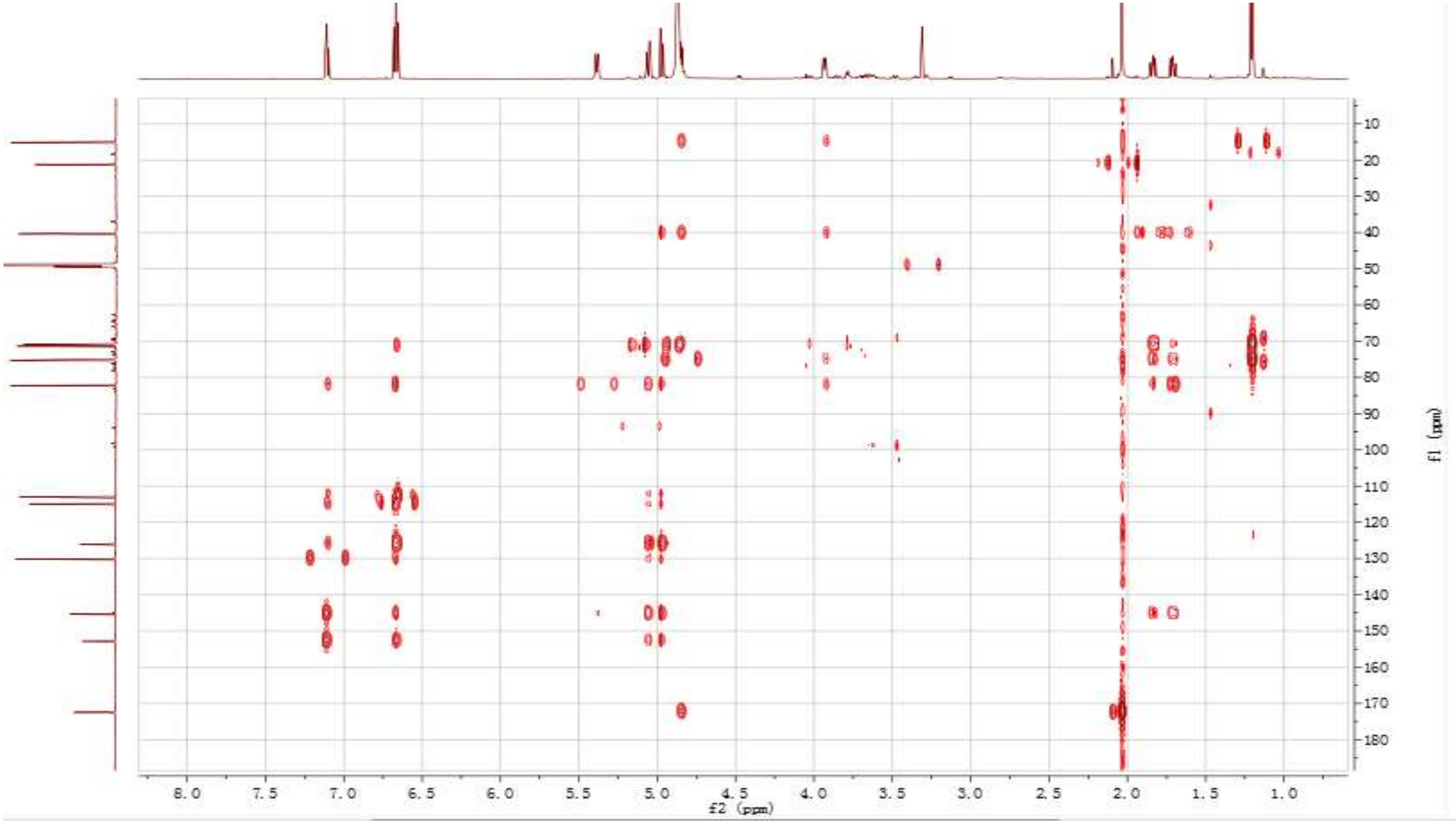

Figure S41. HMBC spectrum of the new compound **5**

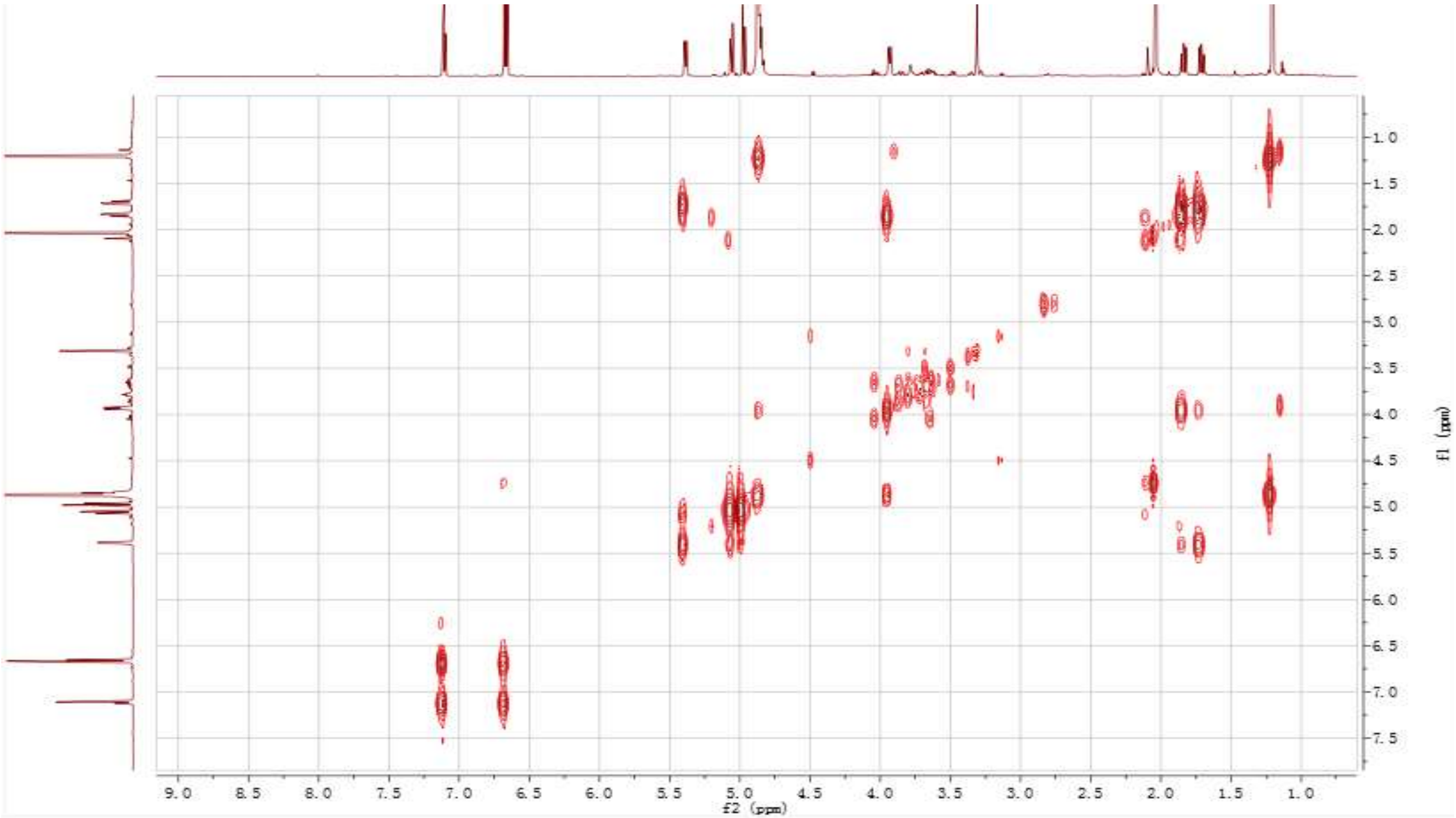

Figure S42. COSY spectrum of the new compound **5**

Mass Spectrum SmartFormula Report

Analysis Info

Analysis Name	D:\Data\MS\data\201901\yangbin_L-41_pos_26_01_6106.d	Acquisition Date	1/17/2019 4:13:43 PM	
Method	LC_Direct Infusion_pos_70-500mz.m	Operator	SCSIO	
Sample Name	yangbin_L-41_pos	Instrument	maXis	255552.00029
Comment				

Acquisition Parameter

Source Type	ESI	Ion Polarity	Positive	Set Nebulizer	0.4 Bar
Focus	Active	Set Capillary	4500 V	Set Dry Heater	180 °C
Scan Begin	70 m/z	Set End Plate Offset	-500 V	Set Dry Gas	4.0 l/min
Scan End	1500 m/z	Set Charging Voltage	0 V	Set Divert Valve	Waste
		Set Corona	0 nA	Set APCI Heater	0 °C

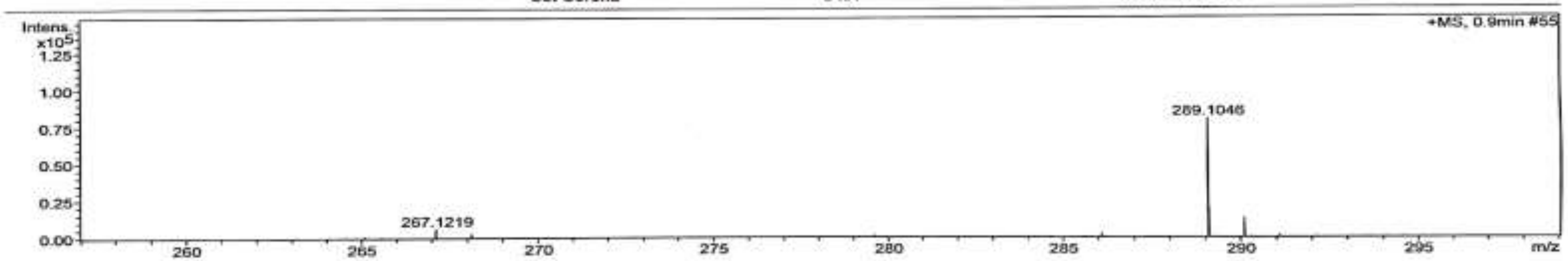

Meas. m/z	#	Ion Formula	Score	m/z	err [ppm]	err [mDa]	mSigma	rdb	e⁻ Conf	N-Rule
267.1219	1	C14H19O5	100.00	267.1227	-3.0	-0.8	69.6	5.5	even	ok
289.1046	1	C14H18NaO5	100.00	289.1046	0.0	0.0	3.4	5.5	even	ok
555.2197	1	C28H36NaO10	100.00	555.2201	0.6	0.4	32.6	10.5	even	ok

yangbin_L-41_pos_26_01_6106.d
Bruker Compass DataAnalysis 4.1 printed: 1/17/2019 4:18:54 PM by: SCSIO Page 1 of 1

Figure S43. HRESI-MS spectrum of compound **6**

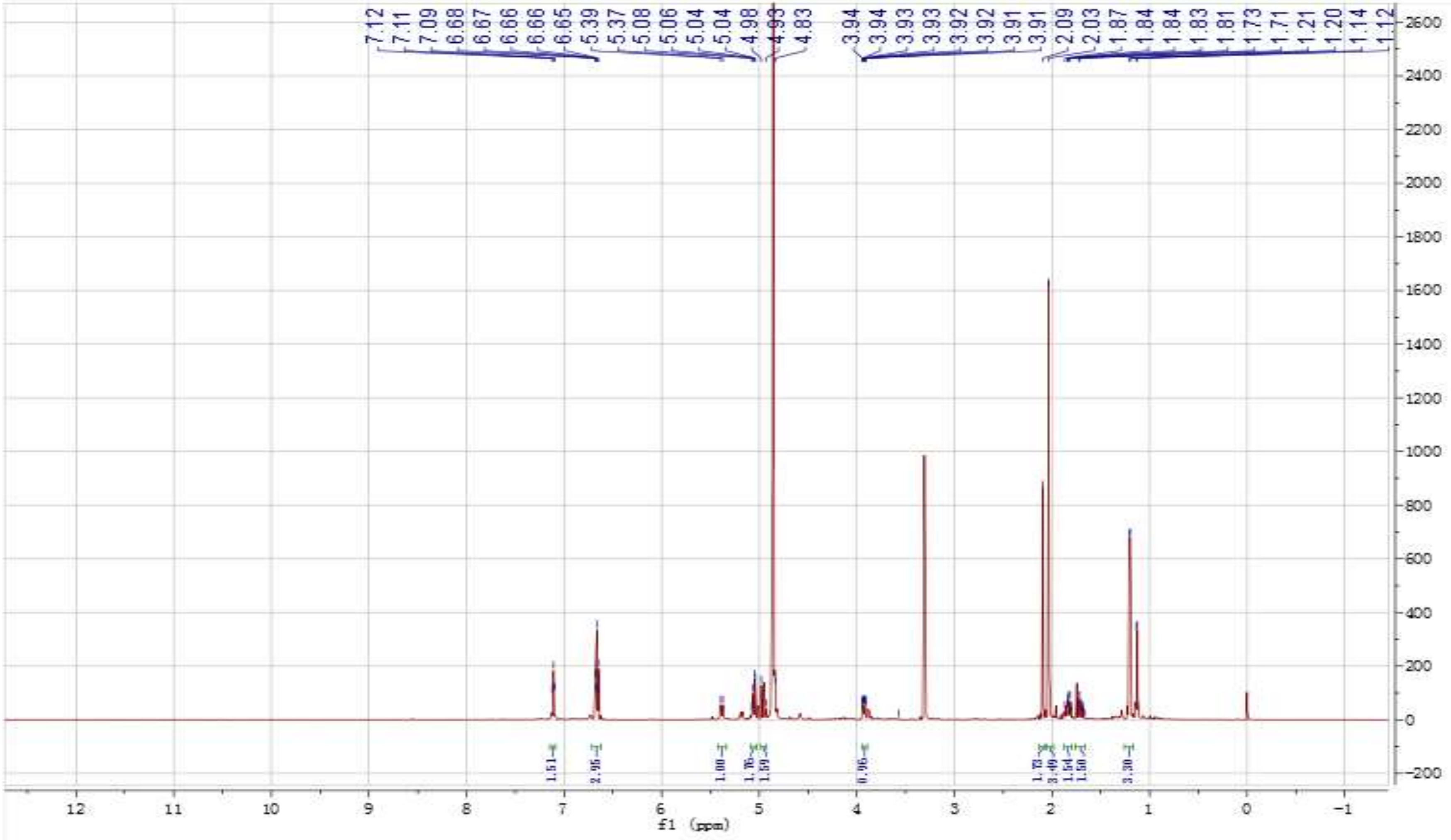

Figure S44. ^{1}H NMR (500 MHz, CD_3OD) spectrum of compound **6**

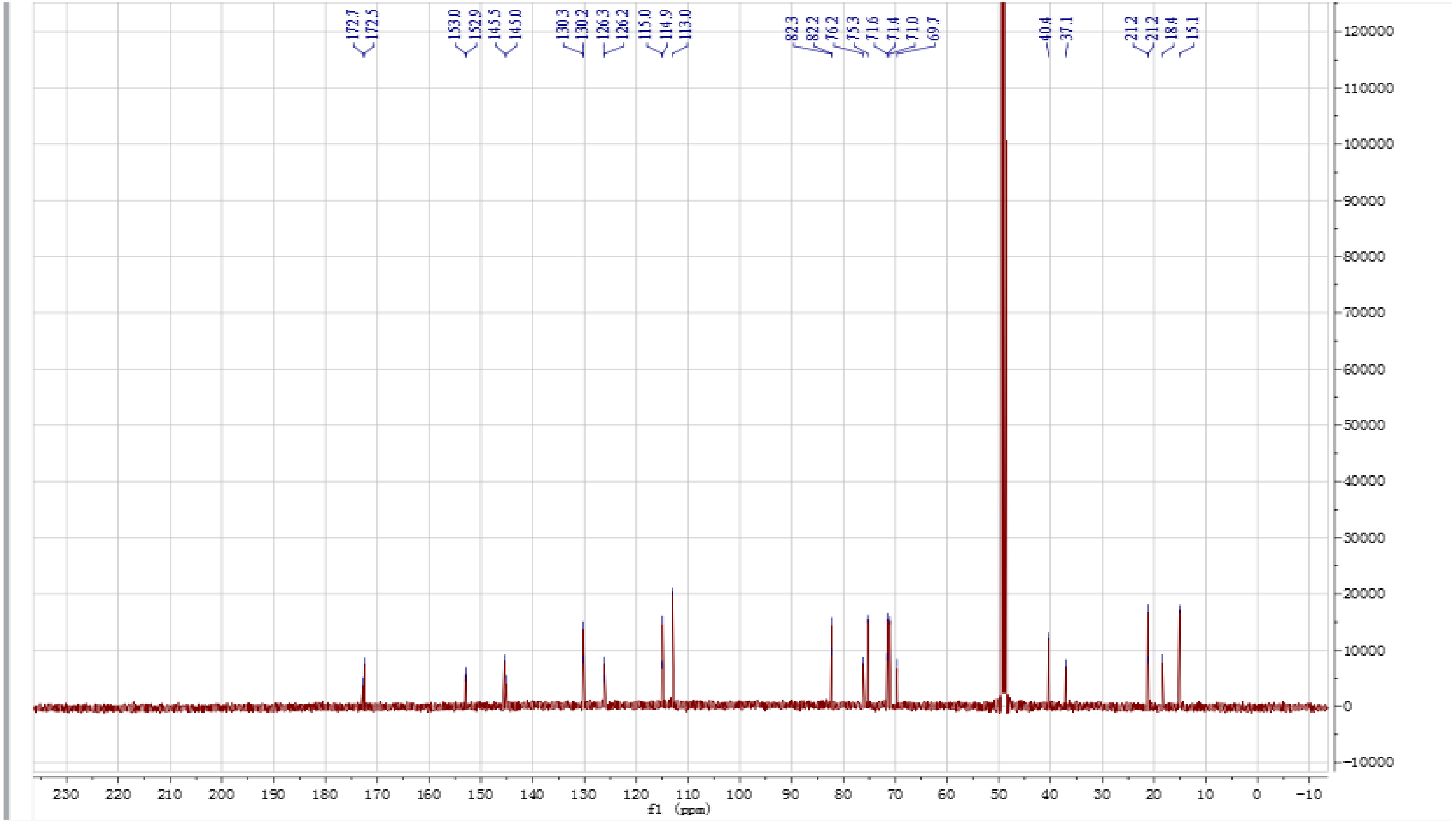

Figure S45. ^{13}C NMR (125 MHz, CD_3OD) spectrum of compound **6**

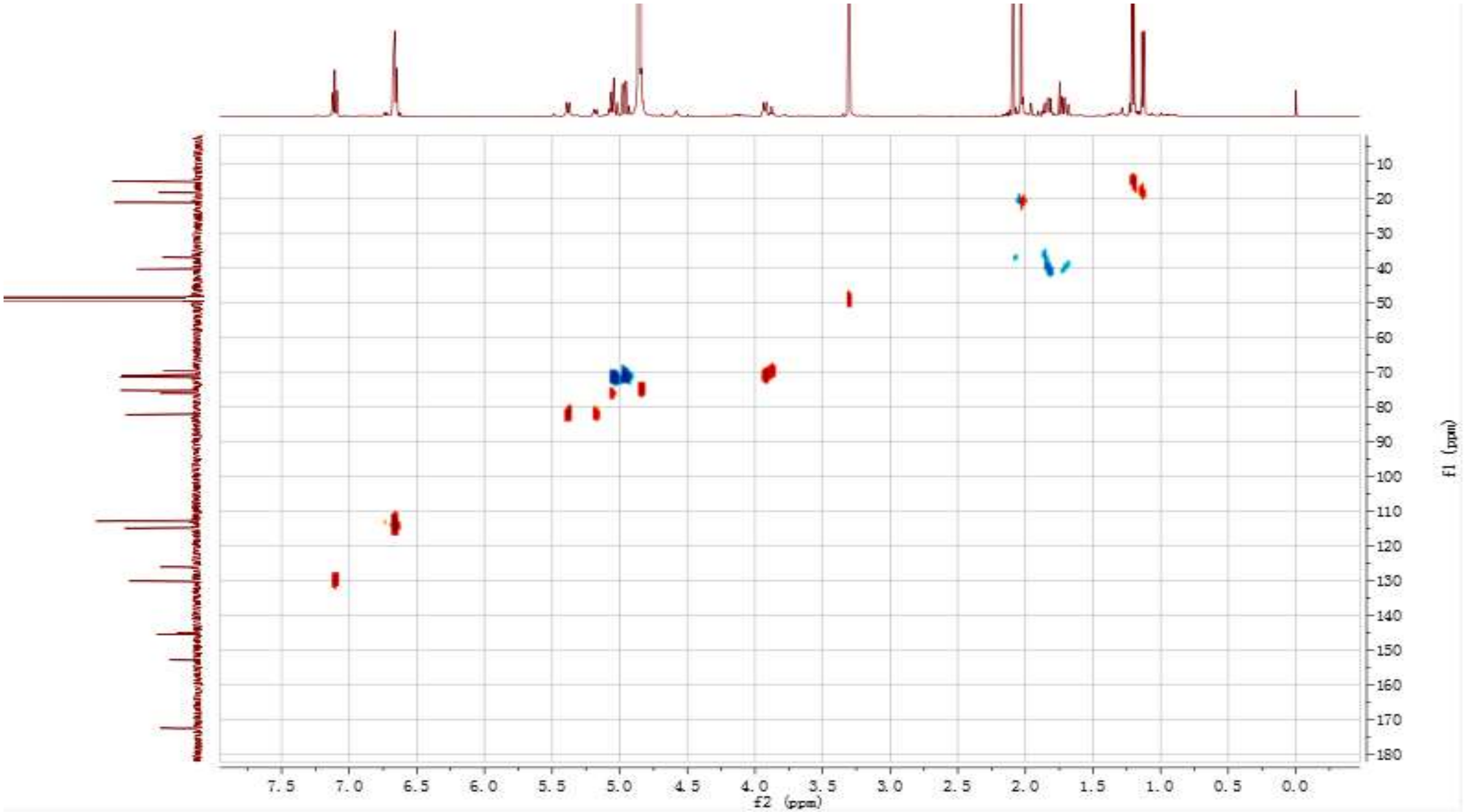

Figure S46. HSQC spectrum of compound **6**

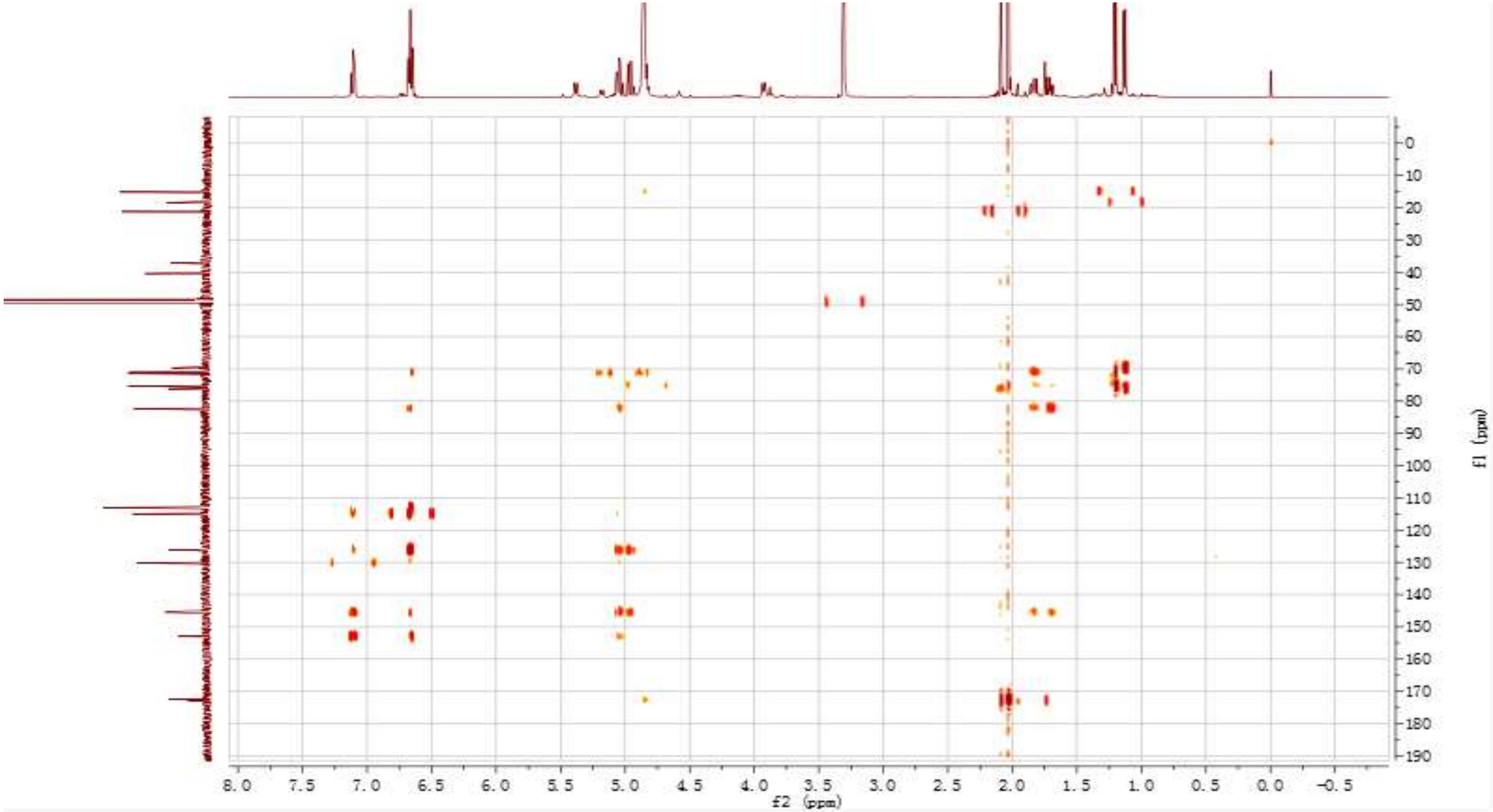

Figure S47. HMBC spectrum of compound **6**

Mass Spectrum SmartFormula Report

Analysis Info

Analysis Name	D:\Data\MS\data\201901\yangbin_L-39_pos_24_01_6104.d	Acquisition Date	1/17/2019 4:06:47 PM
Method	LC_Direct Infusion_pos_70-500mz.m	Operator	SCSIO
Sample Name	yangbin_L-39_pos	Instrument	maXis 255552.00029
Comment			

Acquisition Parameter

Source Type	ESI	Ion Polarity	Positive	Set Nebulizer	0.4 Bar
Focus	Active	Set Capillary	4500 V	Set Dry Heater	180 °C
Scan Begin	70 m/z	Set End Plate Offset	-500 V	Set Dry Gas	4.0 l/min
Scan End	1500 m/z	Set Charging Voltage	0 V	Set Divert Valve	Waste
		Set Corona	0 nA	Set APCI Heater	0 °C

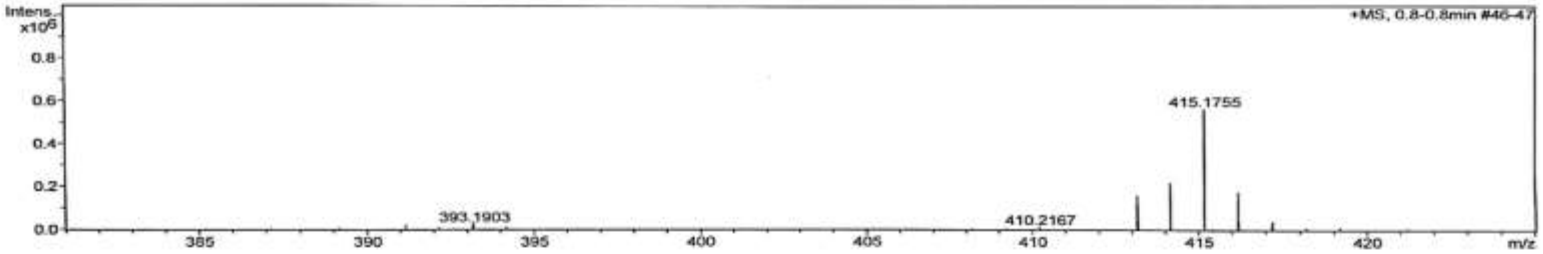

Meas. m/z	#	Ion Formula	Score	m/z	err [ppm]	err [mDa]	mSigma	rdb	e⁻ Conf	N-Rule
391.1760	1	C21H27O7	0.00	391.1751	2.1	0.8	518.3	8.5	even	ok
393.1903	1	C21H29O7	100.00	393.1908	-1.1	-0.4	35.9	7.5	even	ok
413.1580	1	C21H26NaO7	12.15	413.1571	-2.3	-1.0	406.8	8.5	even	ok
415.1755	1	C21H28NaO7	14.56	415.1727	-6.6	-2.8	44.1	7.5	even	ok

yangbin_L-39_pos_24_01_6104.d
Bruker Compass DataAnalysis 4.1 printed: 1/17/2019 4:13:21 PM by: SCSIO Page 1 of 1

Figure S48. HRESI-MS spectrum of the new compound **7**

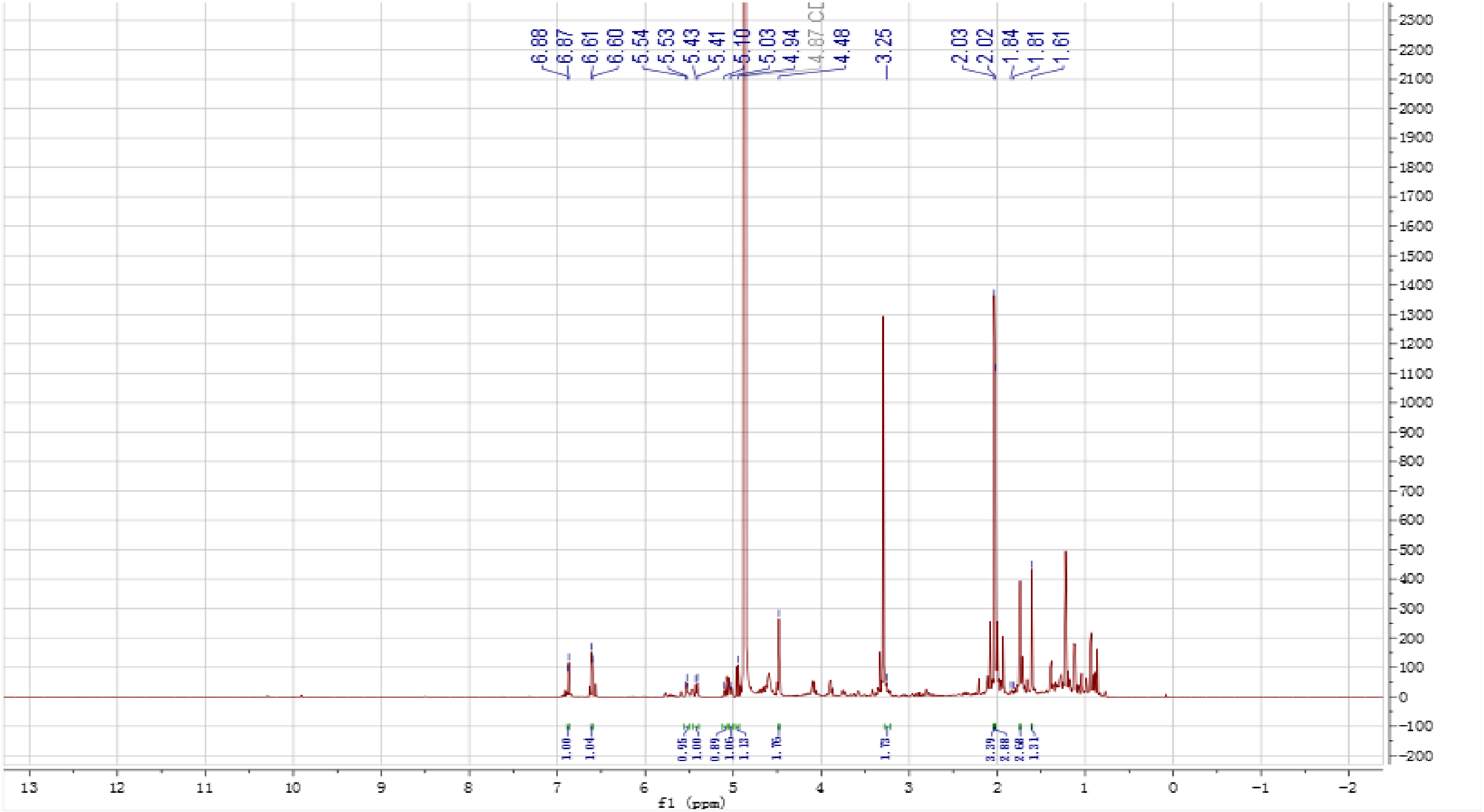

Figure S49. ^{1}H NMR (500 MHz, CD_3OD) spectrum of the new compound **7**

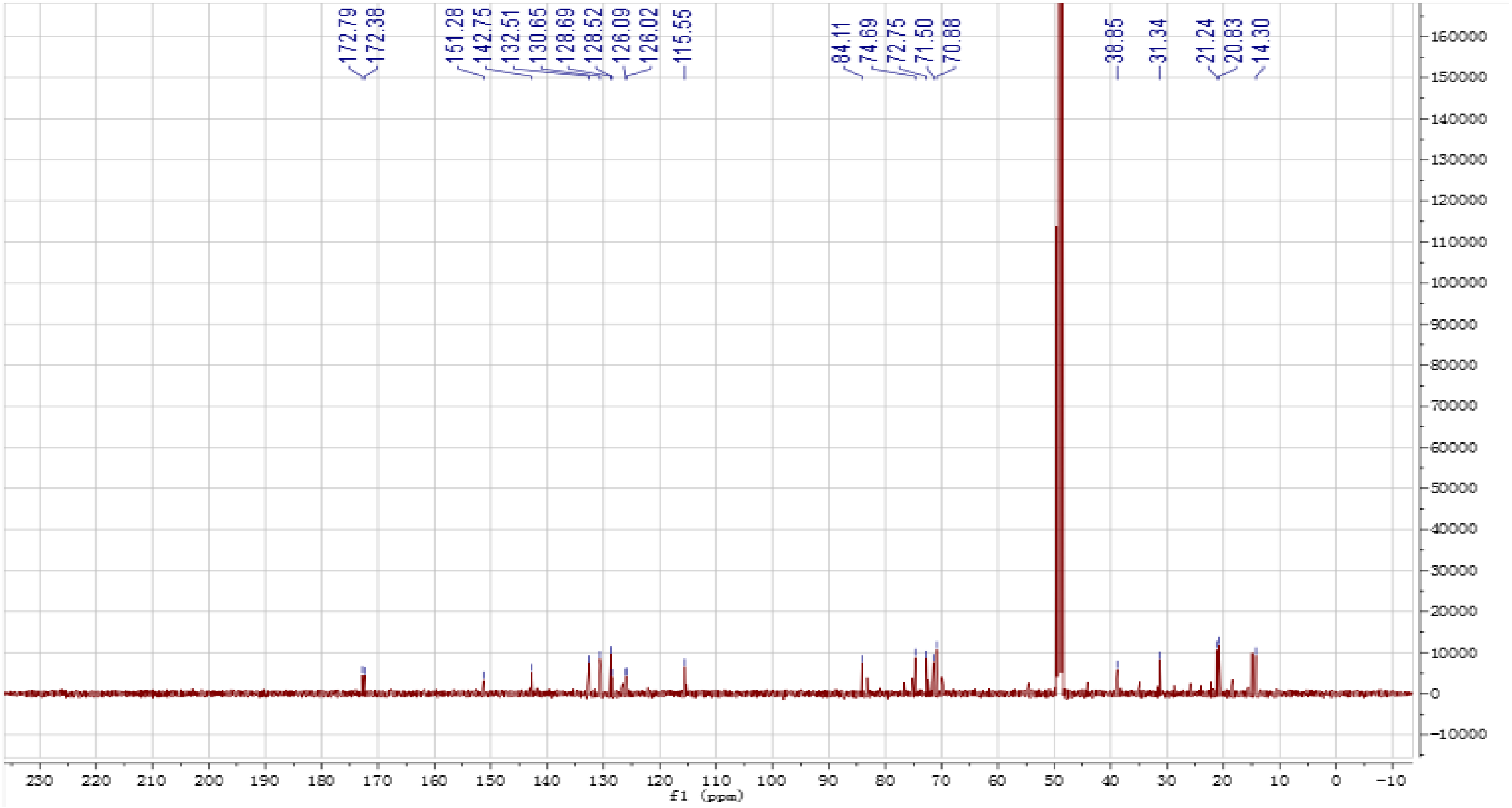

Figure S50. ^{13}C NMR (125 MHz, CD_3OD) spectrum of the new compound **7**

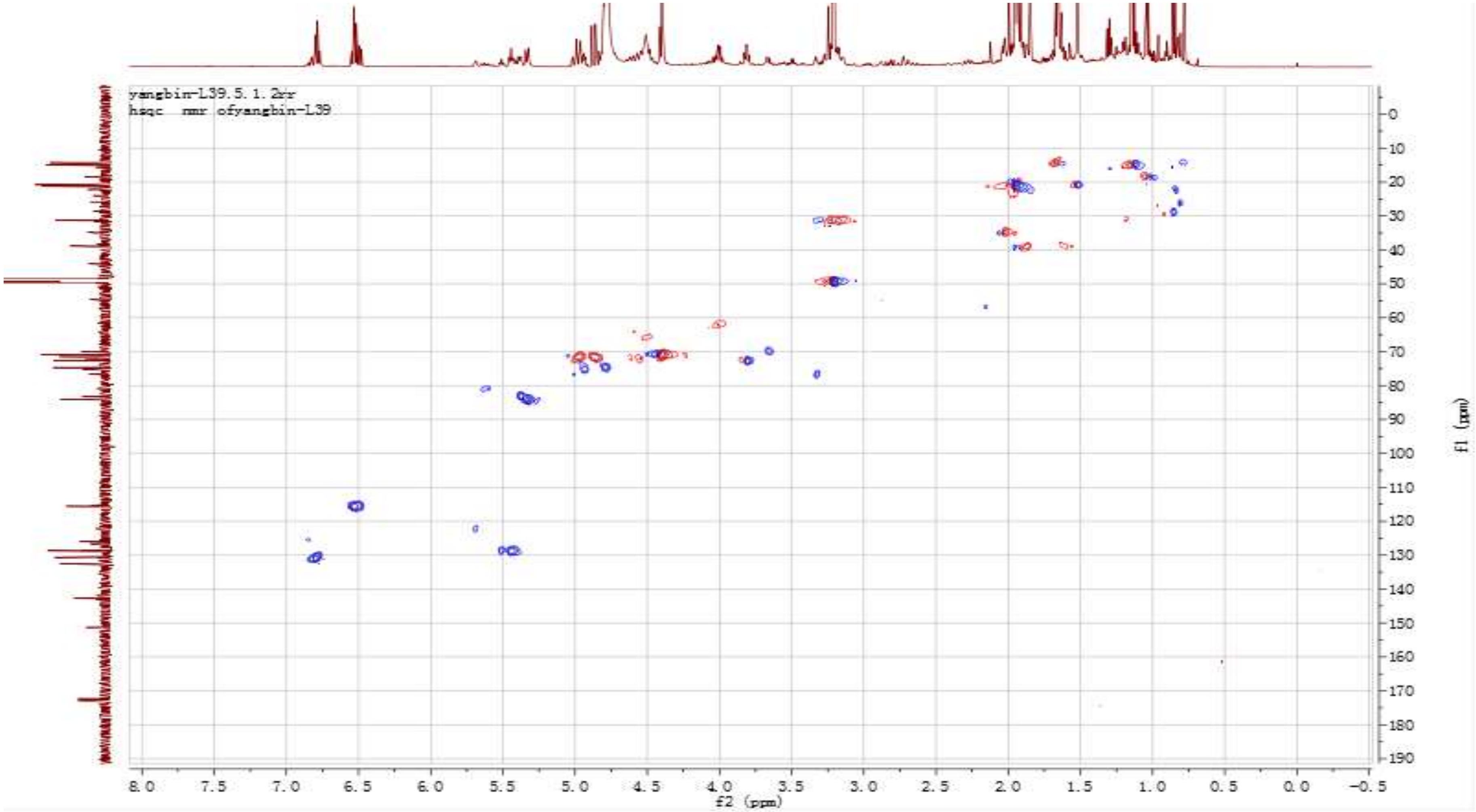

Figure S51. HSQC spectrum of the new compound **7**

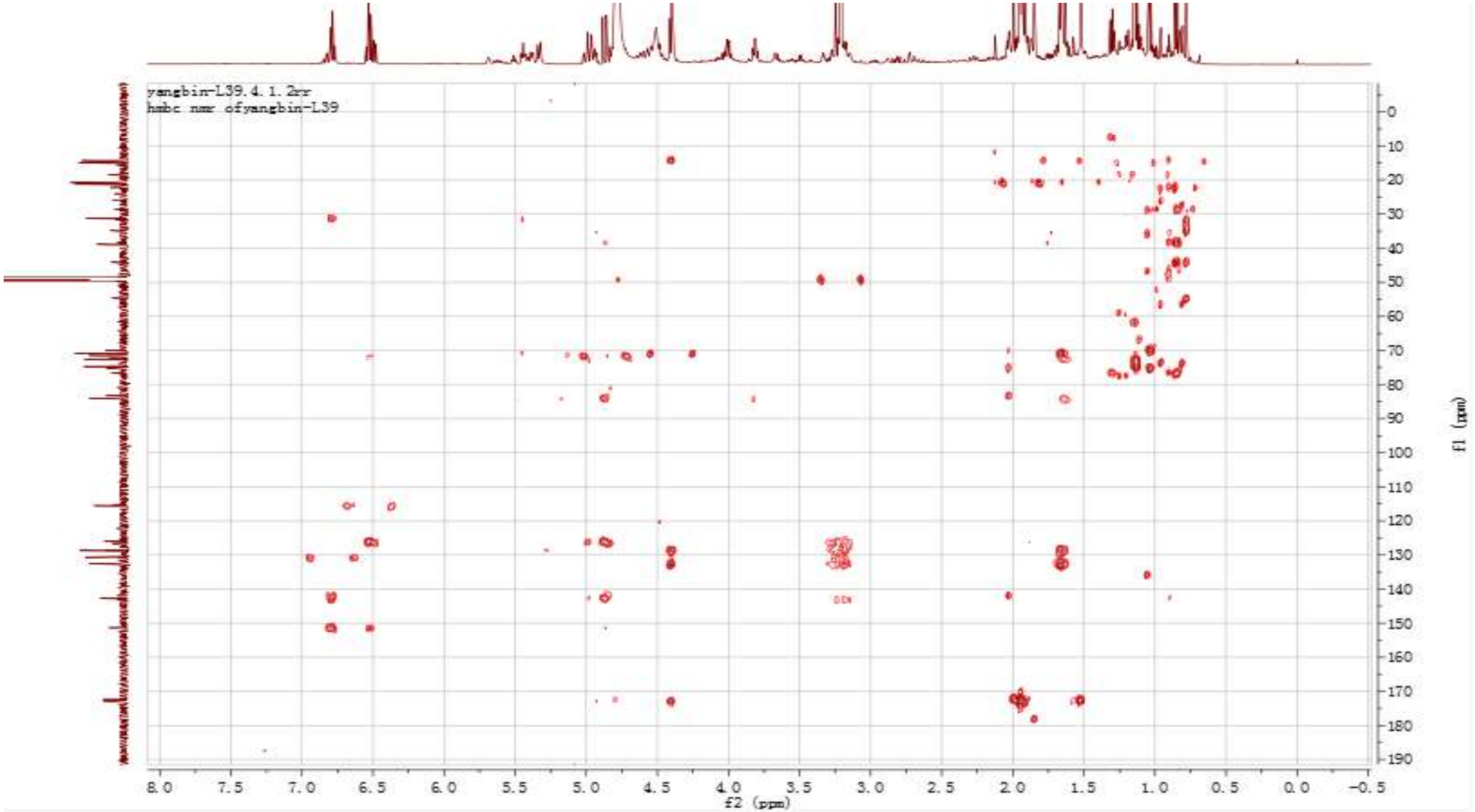

Figure S52. HMBC spectrum of the new compound **7**

Mass Spectrum SmartFormula Report

Analysis Info		Acquisition Date	1/17/2019 4:10:16 PM	
Analysis Name	D:\Data\MS\data\201901\yangbin_L-40_pos_25_01_6105.d			
Method	LC_Direct Infusion_pos_70-500mz.m	Operator	SCSIO	
Sample Name	yangbin_L-40_pos	Instrument	maXis	255552.00029
Comment				

Acquisition Parameter

Source Type	ESI	Ion Polarity	Positive	Set Nebulizer	0.4 Bar
Focus	Active	Set Capillary	4500 V	Set Dry Heater	180 °C
Scan Begin	70 m/z	Set End Plate Offset	-500 V	Set Dry Gas	4.0 l/min
Scan End	1500 m/z	Set Charging Voltage	0 V	Set Divert Valve	Waste
		Set Corona	0 nA	Set APCI Heater	0 °C

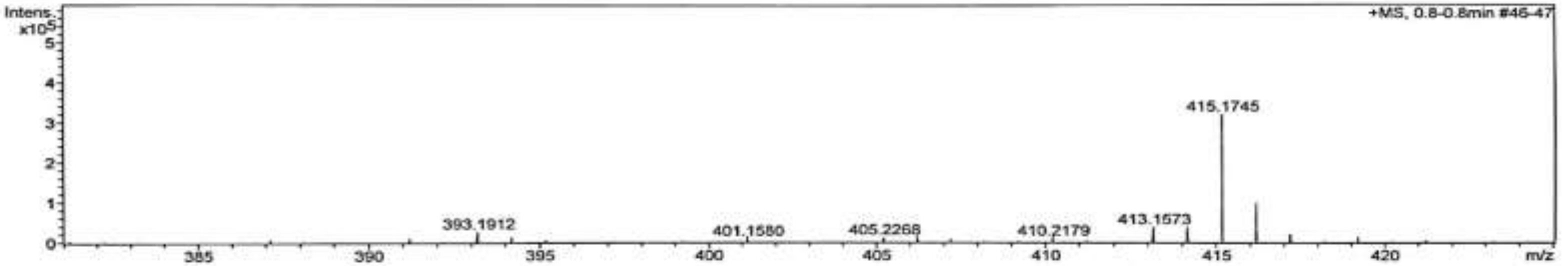

Meas. m/z	#	Ion Formula	Score	m/z	err [ppm]	err [mDa]	mSigma	rdb	e⁻ Conf	N-Rule
393.1912	1	C21H29O7	100.00	393.1908	-1.0	-0.4	65.3	7.5	even	ok
415.1745	1	C21H28NaO7	35.20	415.1727	-4.3	-1.8	37.3	7.5	even	ok
805.3423	1	C42H54NaO14	100.00	805.3406	-2.2	-1.8	602.9	15.5	even	ok
807.3575	1	C42H56NaO14	100.00	807.3562	-1.5	-1.2	27.9	14.5	even	ok

yangbin_L-40_pos_25_01_6105.d

Bruker Compass DataAnalysis 4.1 printed: 1/17/2019 4:15:57 PM by: SCSIO Page 1 of 1

Figure S53. HRESI-MS spectrum of the new compound **8**

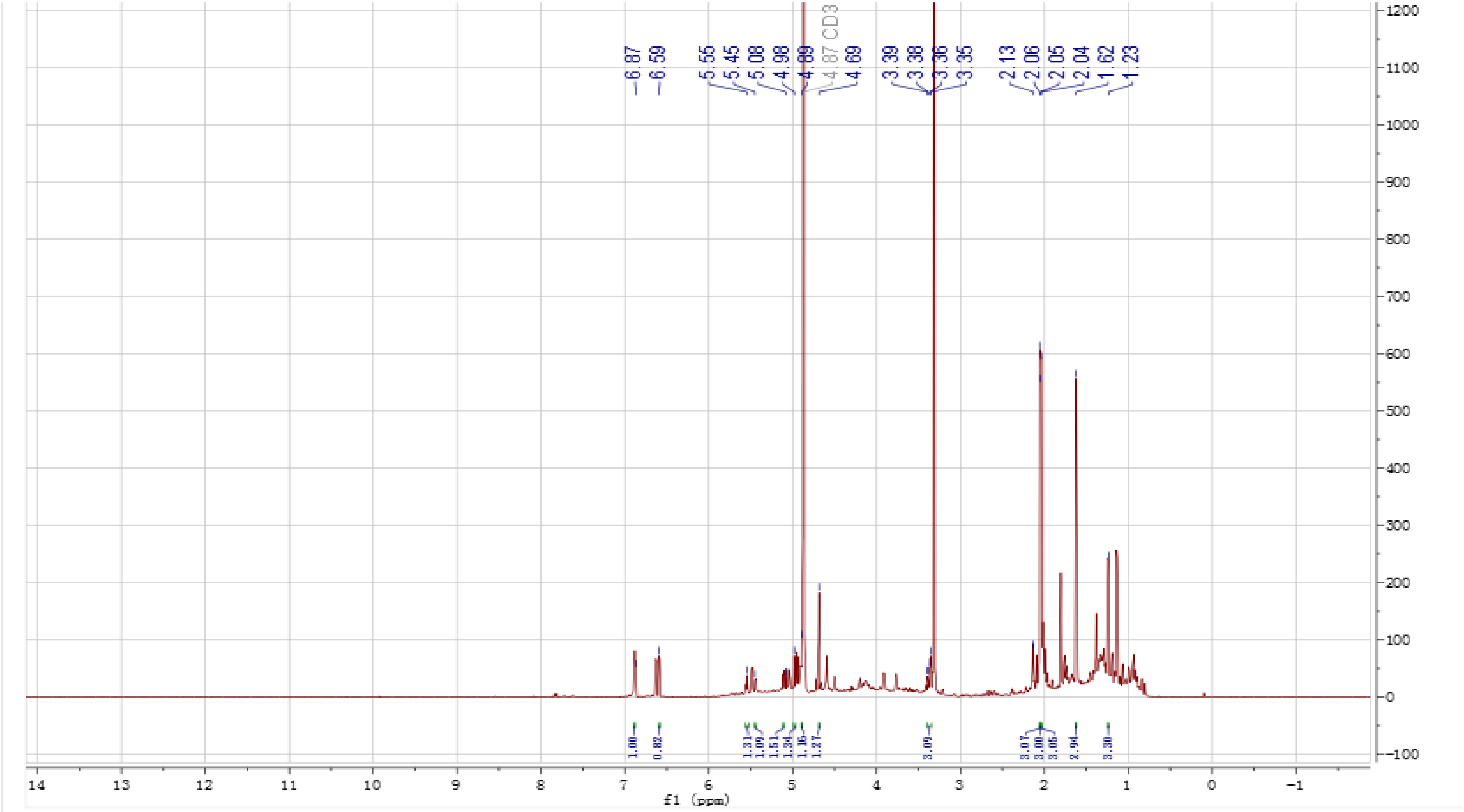

Figure S54. ^{1}H NMR (500 MHz, CD_3OD) spectrum of the new compound **8**

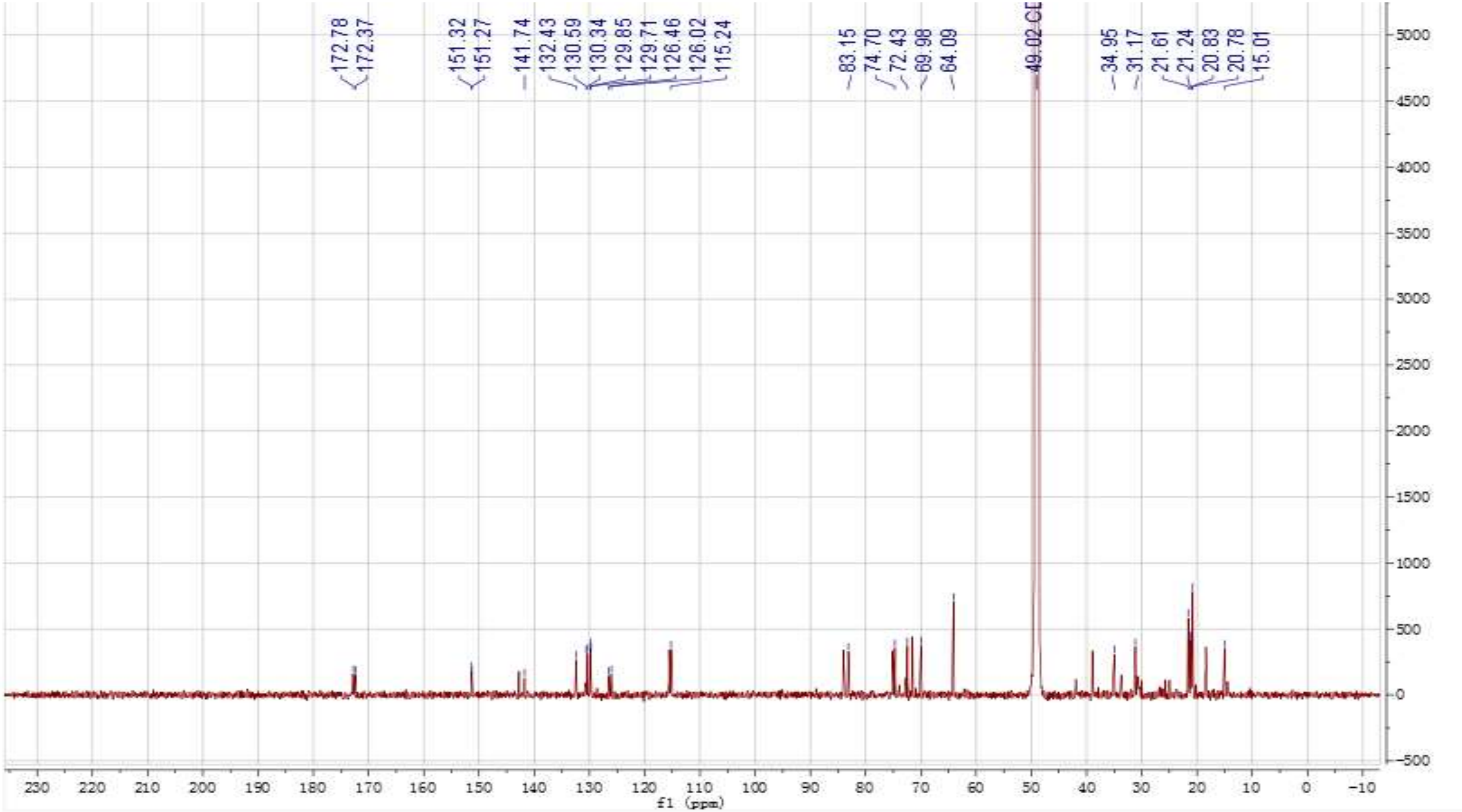

Figure S55. ^{13}C NMR (125 MHz, CD_3OD) spectrum of the new compound **8**

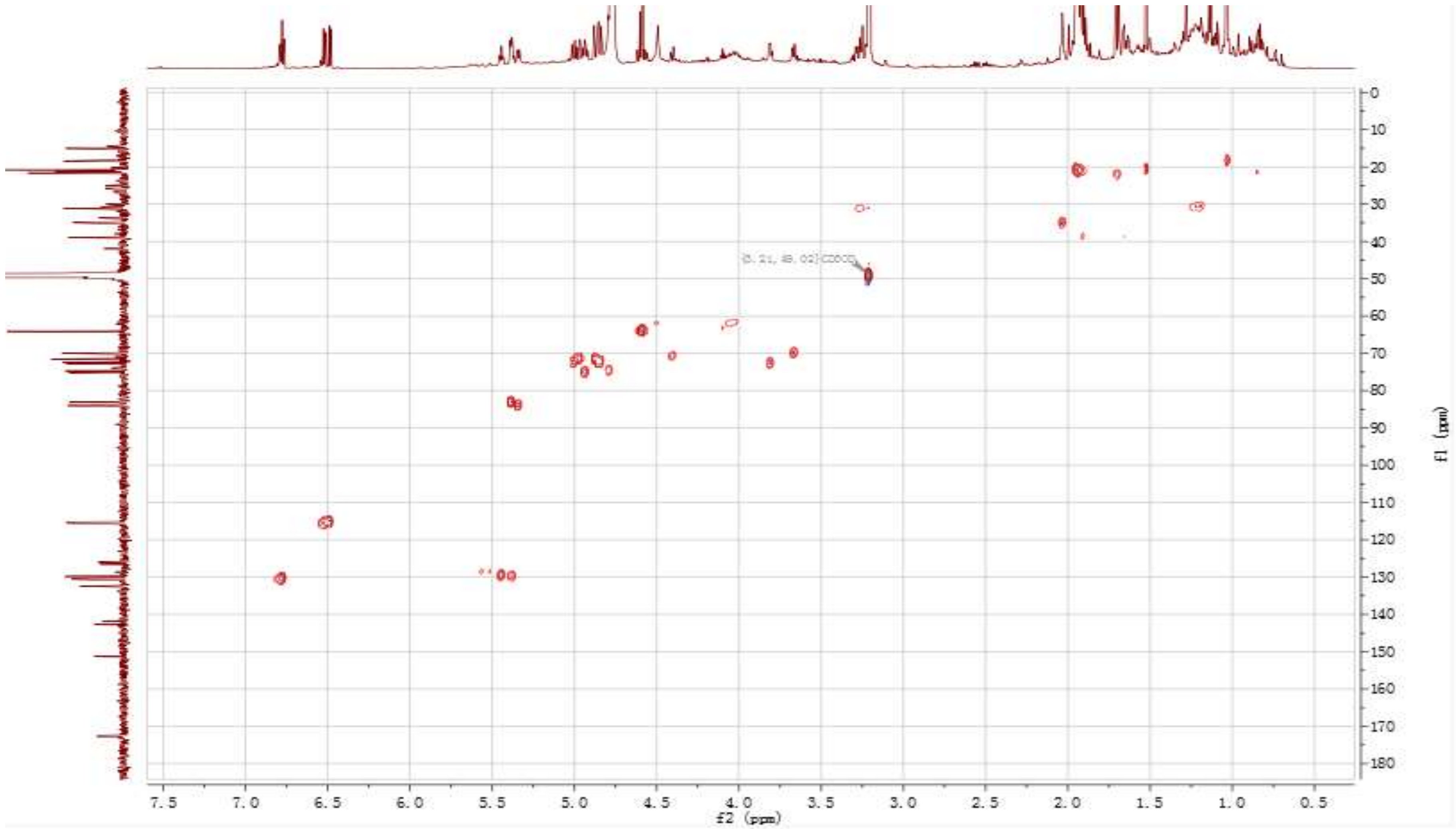

Figure S56. HSQC spectrum of the new compound **8**

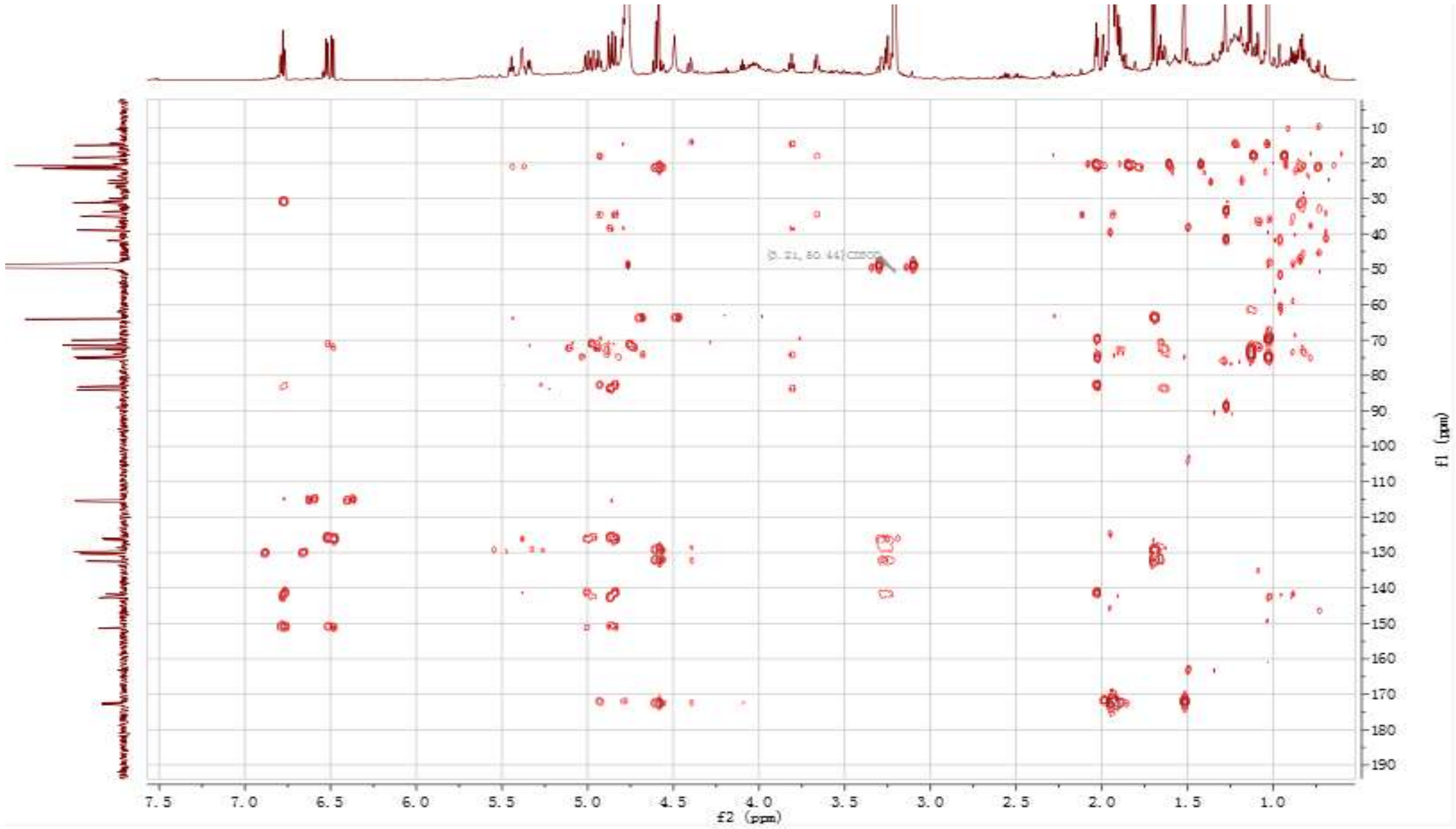

Figure S57. HMBC spectrum of the new compound **8**

Mass Spectrum SmartFormula Report

Analysis Info

Analysis Name	D:\Data\MS\data\201812\yangbin_L28_pos_31_01_6015.d	Acquisition Date	12/27/2018 5:21:48 PM	
Method	LC_Direct Infusion_pos_70-500mz.m	Operator	SCSIO	
Sample Name	yangbin_L28_pos	Instrument	maXis	255552.00029
Comment				

Acquisition Parameter

Source Type	ESI	Ion Polarity	Positive	Set Nebulizer	0.4 Bar
Focus	Active	Set Capillary	4500 V	Set Dry Heater	180 °C
Scan Begin	70 m/z	Set End Plate Offset	-500 V	Set Dry Gas	4.0 l/min
Scan End	1500 m/z	Set Charging Voltage	0 V	Set Divert Valve	Waste
		Set Corona	0 nA	Set APCI Heater	0 °C

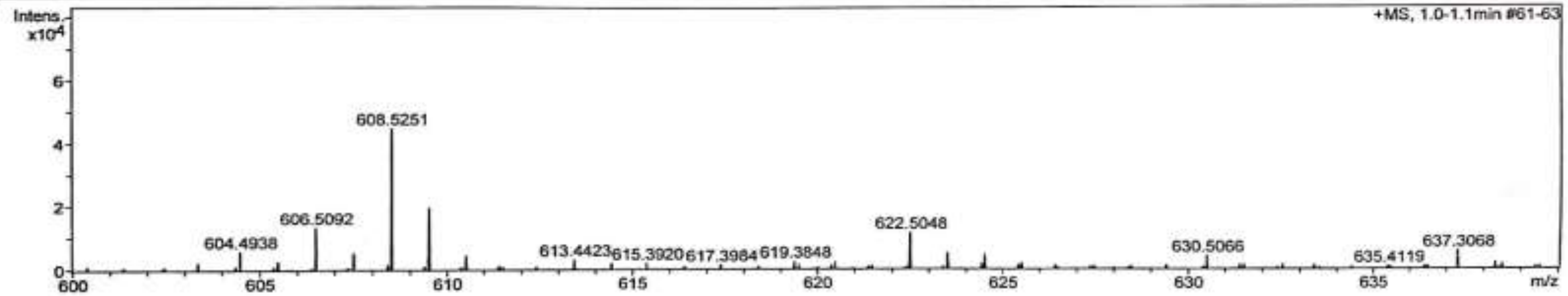

Meas. m/z	#	Ion Formula	Score	m/z	err [ppm]	err [mDa]	mSigma	rdb	e⁻ Conf	N-Rule
608.5251	1	C37H70NO5	100.00	608.5249	-0.4	-0.3	13.9	3.5	even	ok
630.5066	1	C37H69NNaO5	100.00	630.5068	0.3	0.2	51.9	3.5	even	ok
1216.0430	1	C74H139N2O10	100.00	1216.0424	0.4	0.5	20.3	6.5	even	ok

yangbin_L28_pos_31_01_6015.d

Bruker Compass DataAnalysis 4.1 printed: 12/27/2018 5:33:06 PM by: SCSIO Page 1 of 1

Figure S58. HRESI-MS spectrum of the new compound **9**

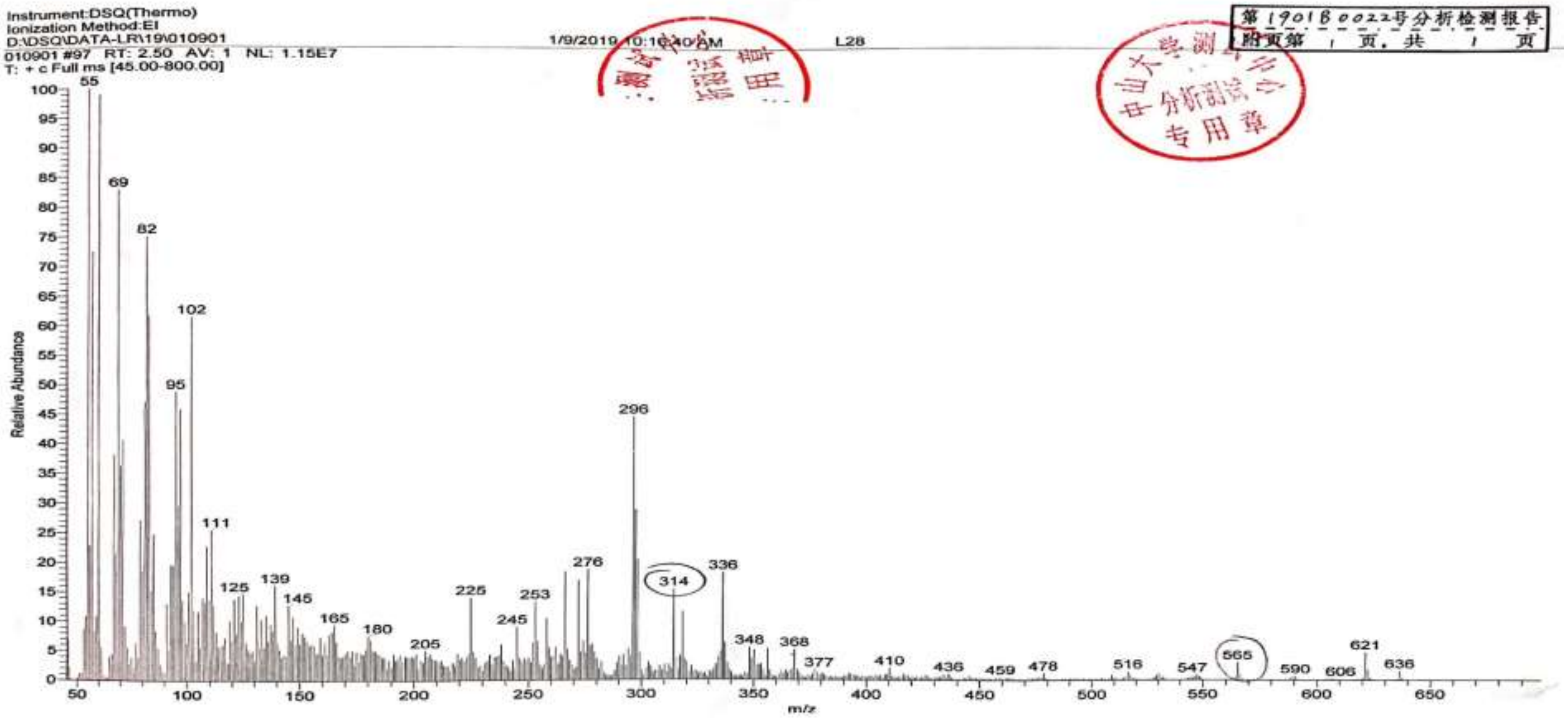

Figure S59. EI-MS spectrum of the new compound **9**

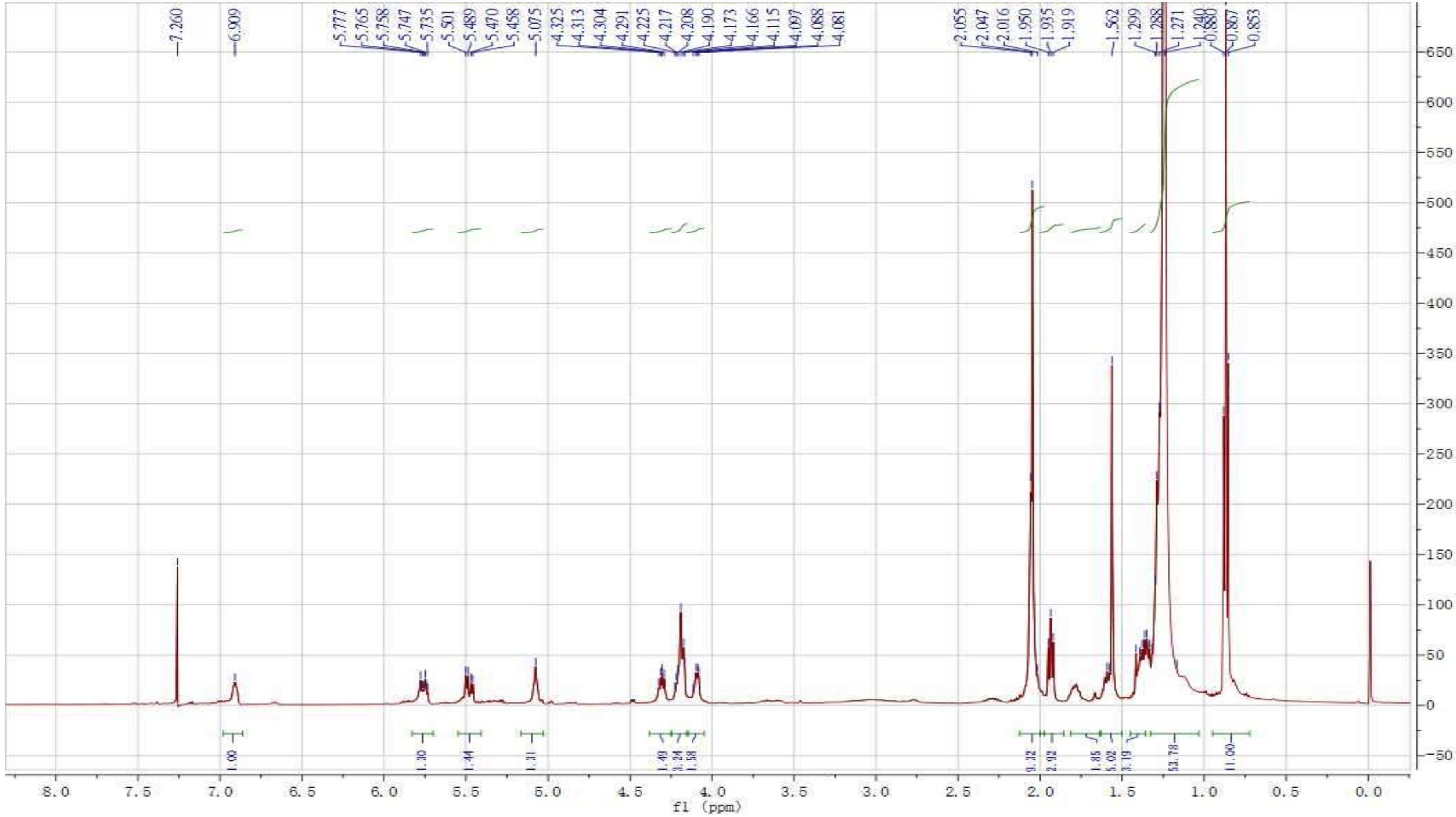

Figure S60. ^{1}H NMR (500 MHz, $CDCl_3$) spectrum of the new compound **9**

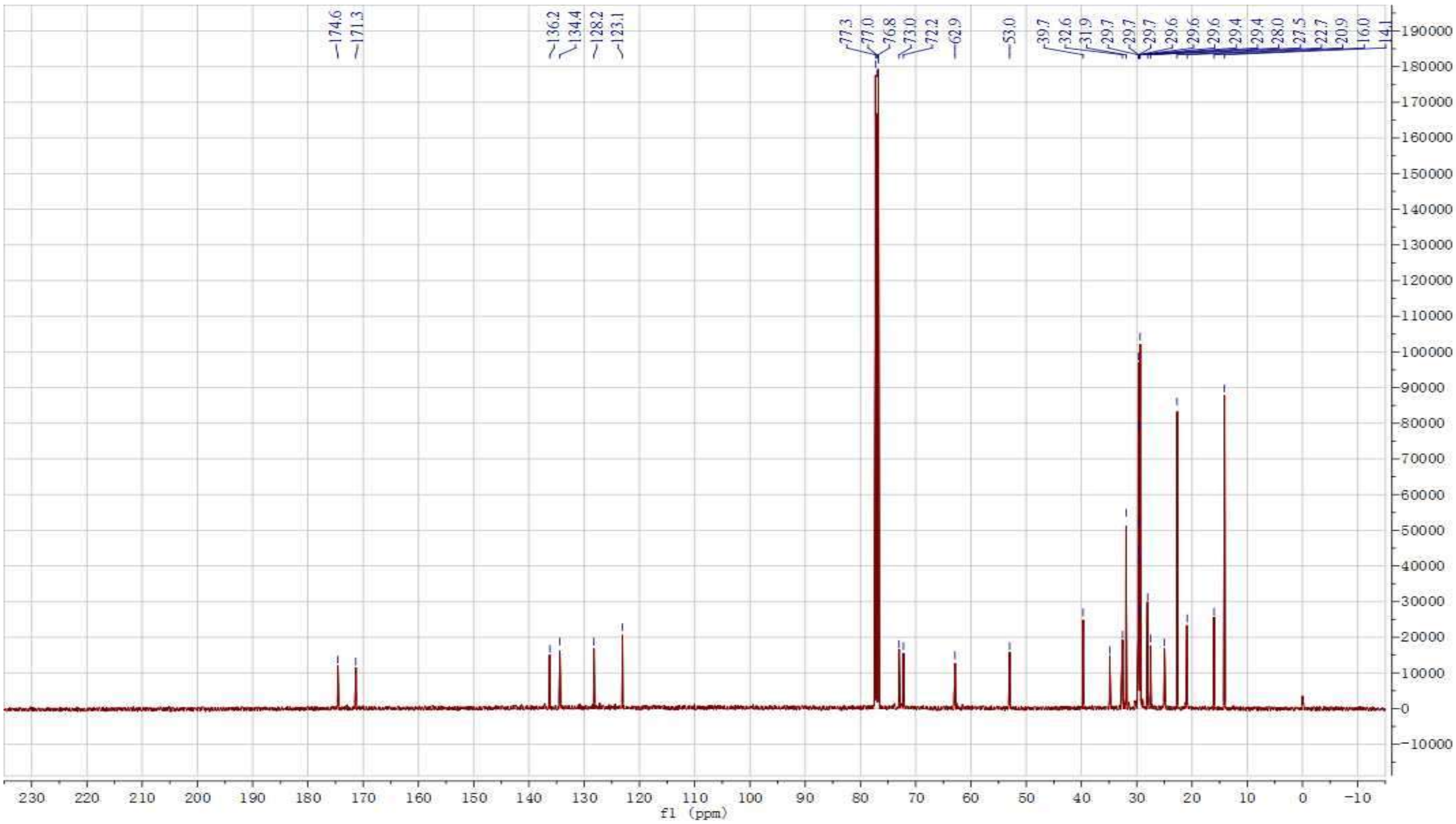

Figure S61. ^{13}C NMR (125 MHz, $CDCl_3$) spectrum of the new compound **9**

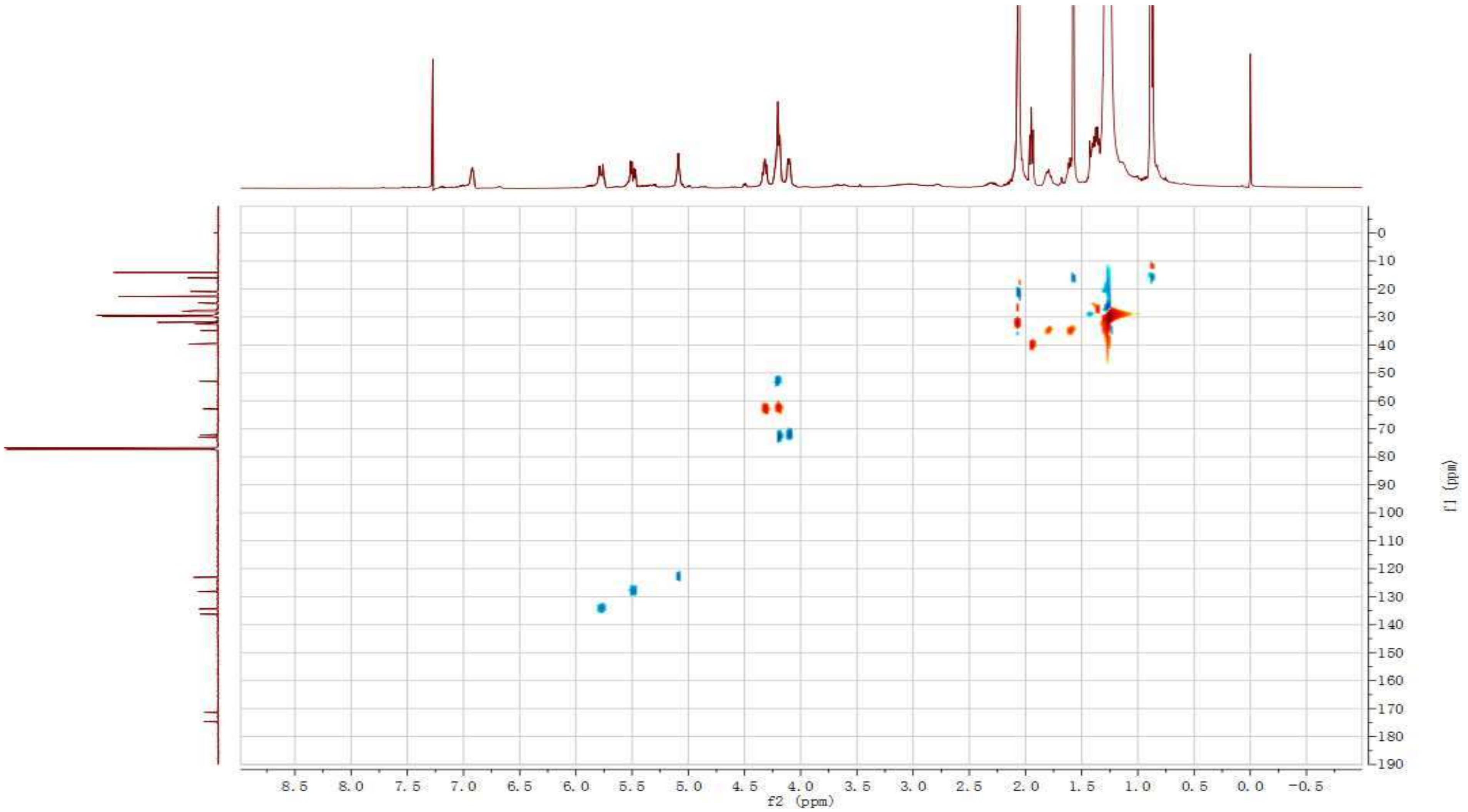

Figure S62. HSQC spectrum of the new compound **9**

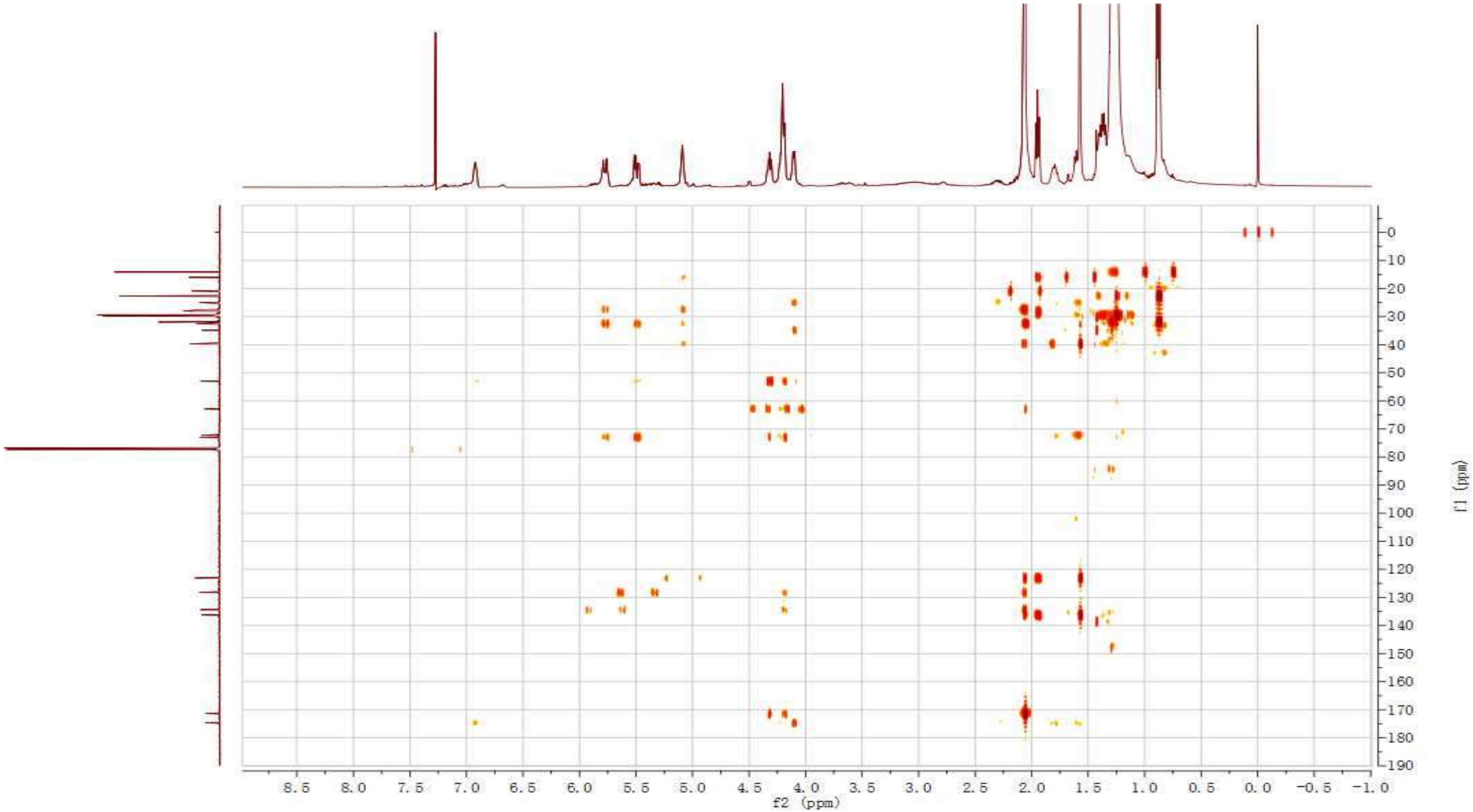

Figure S63. HMBC spectrum of the new compound **9**

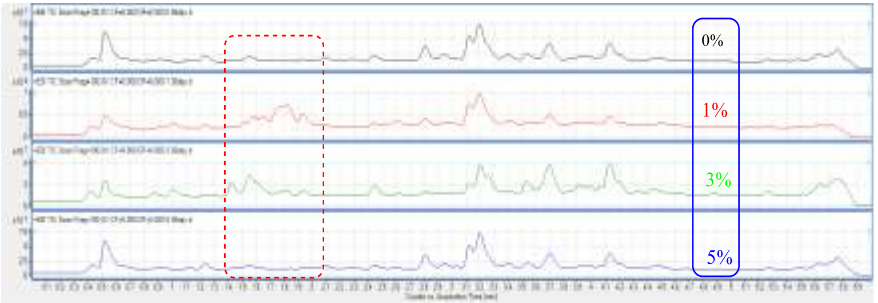

Figure S64. HPLC chromatograms of EtOAc extracts monitored (black line: 0% NaCl rice medium, red line: 1% NaCl rice medium, green line: 3% NaCl rice medium, blue line: 5% NaCl rice medium).
